# Prion acute synaptotoxicity is largely driven by protease-resistant PrP^Sc^ species

**DOI:** 10.1371/journal.ppat.1007214

**Published:** 2018-08-08

**Authors:** Simote Totauhelotu Foliaki, Victoria Lewis, David Isaac Finkelstein, Victoria Lawson, Harold Arthur Coleman, Matteo Senesi, Abu Mohammed Taufiqual Islam, Feng Chen, Shannon Sarros, Blaine Roberts, Paul Anthony Adlard, Steven John Collins

**Affiliations:** 1 Department of Medicine (Royal Melbourne Hospital), The University of Melbourne, Parkville, Victoria, Australia; 2 Florey Institute of Neuroscience and Mental Health, Parkville, Victoria, Australia; 3 Department of Pathology, The University of Melbourne, Parkville, Victoria, Australia; 4 Department of Physiology, Monash University, Clayton, Victoria, Australia; 5 Monash Biomedicine Discovery Institute, Monash University, Clayton, Victoria, Australia; University of Alberta, CANADA

## Abstract

Although misfolding of normal prion protein (PrP^C^) into abnormal conformers (PrP^Sc^) is critical for prion disease pathogenesis our current understanding of the underlying molecular pathophysiology is rudimentary. Exploiting an electrophysiology paradigm, herein we report that at least modestly proteinase K (PK)-resistant PrP^Sc^ (PrP^res^) species are acutely synaptotoxic. Brief exposure to *ex vivo* PrP^Sc^ from two mouse-adapted prion strains (M1000 and MU02) prepared as crude brain homogenates (cM1000 and cMU02) and cell lysates from chronically M1000-infected RK13 cells (MoRK13-Inf) caused significant impairment of hippocampal CA1 region long-term potentiation (LTP), with the LTP disruption approximating that reported during the evolution of murine prion disease. Proof of PrP^Sc^ (especially PrP^res^) species as the synaptotoxic agent was demonstrated by: significant rescue of LTP following selective immuno-depletion of total PrP from cM1000 (dM1000); modestly PK-treated cM1000 (PK+M1000) retaining full synaptotoxicity; and restoration of the LTP impairment when employing reconstituted, PK-eluted, immuno-precipitated M1000 preparations (PK+IP-M1000). Additional detailed electrophysiological analyses exemplified by impairment of post-tetanic potentiation (PTP) suggest possible heightened pre-synaptic vulnerability to the acute synaptotoxicity. This dysfunction correlated with cumulative insufficiency of replenishment of the readily releasable pool (RRP) of vesicles during repeated high-frequency stimulation utilised for induction of LTP. Broadly comparable results with LTP and PTP impairment were obtained utilizing hippocampal slices from PrP^C^ knockout (PrPo/o) mice, with cM1000 serial dilution assessments revealing similar sensitivity of PrPo/o and wild type (WT) slices. Size fractionation chromatography demonstrated that synaptotoxic PrP correlated with PK-resistant species >100kDa, consistent with multimeric PrP^Sc^, with levels of these species >6 ng/ml appearing sufficient to induce synaptic dysfunction. Biochemical analyses of hippocampal slices manifesting acute synaptotoxicity demonstrated reduced levels of multiple key synaptic proteins, albeit with noteworthy differences in PrPo/o slices, while such changes were absent in hippocampi demonstrating rescued LTP through treatment with dM1000. Our findings offer important new mechanistic insights into the synaptic impairment underlying prion disease, enhancing prospects for development of targeted effective therapies.

## Author summary

Misfolding of the normal prion protein (PrP^C^) into disease-associated conformations (PrP^Sc^) is the critical initiating step for prion diseases. Similar to other neurodegenerative disorders, progressive failure of brain synapses is considered a primary deleterious event underpinning prion disease evolution. Our current understanding of the underlying mechanisms associated with synaptic failure is rudimentary contributing to difficulties in developing effective treatments. Herein we report the use of an electrophysiology paradigm that allowed us to demonstrate that at least modestly proteinase K (PK)-resistant PrP^Sc^ species from two mouse-adapted prion strains (M1000 and MU02) are directly synaptotoxic causing significant acute impairment of hippocampal CA1 region long-term potentiation (LTP). Of note, the LTP disruption approximated that reported in prion animal models. Additional detailed analyses provided novel pathophysiological insights suggesting possible heightened pre-synaptic vulnerability to the acute synaptotoxicity through impairment of replenishment of the readily releasable pool of neurotransmitter vesicles, while biochemical analyses demonstrated reduced levels of multiple key pre-and post-synaptic proteins. Broadly similar acute synaptic dysfunction and dose-response susceptibility were observed in slices from mice not expressing PrP^C^ albeit with minor but noteworthy differences in electrophysiological and biochemical findings. Our study offers important new mechanistic insights into the synaptic impairment underlying prion disease, enhancing prospects for development effective therapies.

## Introduction

Prion diseases constitute a group of transmissible neurodegenerative disorders with the spectrum encompassing several human phenotypes, the most common being Creutzfeldt-Jakob disease (CJD), as well as a number of animal diseases including bovine spongiform encephalopathy (“mad cow” disease) and scrapie in sheep [1, 2]. Regardless of disease phenotype, misfolding of PrP^C^ into disease-associated conformers (herein collectively designated PrP^Sc^), with their subsequent aggregation and accumulation, appears critical to pathogenesis although the precise neurotoxic species and how such species provoke neuronal dysfunction and loss leading to the onset of clinical illness remain unresolved. The precise composition of the infectious unit or “prion” also remains to be determined, although considerable evidence supports that PrP^Sc^ is the major, if not exclusive, component (the “protein only” hypothesis) [3]. Historically, PrP^Sc^ has been considered to be highly protease-resistant (designated PrP^res^ after protease treatment) but recent evidence supports the existence of a broader spectrum, including protease-sensitive conformers, which most likely contribute to pathogenesis and may comprise up to 90% of misfolded prion protein in diseased brains [4, 5].

The primary function of PrP^C^ in the central nervous system remains uncertain although a key role for this glycosylphosphatidylinositol-anchored glycoprotein in synaptic physiology and memory has been described [6]. Aligned to such functions, PrP^C^ has been reported as having a predominant synaptic localisation [7], with important influences on voltage-gated calcium (Ca^2+^) [8] and N-methyl-D-aspartate receptor (NMDAR) ion channels [9], as well as LTP [10]. LTP is a use-dependent neurophysiological process, enhancing the strength of synaptic connection, with hippocampal CA1 region LTP directly correlating with episodic memory acquisition [11]. Critical to LTP-type synaptic plasticity and episodic memory generated in the hippocampus are α-amino-3-hydroxy-5-methyl-4-isoxazoleproprionic acid receptor (AMPAR) and NMDAR ion channels, as well as metabotropic glutamate receptors with signal transduction mediated through pathways including calcium-regulated phosphorylated extracellular signal–regulated kinase (pERK) and phosphorylated cAMP response element binding protein (pCREB), which alter DNA transcription with consequent ultrastructural and receptor changes at synapses [12].

Our group [13] and others [14, 15] have shown in prion animal models evidence of early selective hippocampal damage, with synapses becoming significantly disrupted and retracted from the mid-incubation period [15–17]. Of particular relevance, selective and progressive impairment of LTP in the CA1 region of the hippocampal stratum radiatum has been demonstrated *in vivo* in ME7 prion infected mice from 44–70% of the incubation period [14], with early loss of hippocampal pyramidal neuronal synapses shown to correlate with first evidence of disturbances in hippocampal-dependent behaviour [15]. Additionally, studies have revealed that impairment of LTP coincides with the earliest detection of PrP^Sc^, slightly prior to morphological evidence of synaptic loss or neuropil vacuolation [14, 18], indicating that impairment of hippocampal CA1 region LTP is a sensitive indicator of synaptic dysfunction in prion strains that cause early, prominent hippocampal damage and supporting the likelihood that PrP^Sc^ is directly synaptotoxic.

Somewhat limiting our ability to better understand prion pathogenesis is the relative paucity of tractable, authentic models of acute prion neurotoxicity. Very simple *in vitro* cell culture models have demonstrated toxic effects of recombinant, soluble, oligomeric PrP enriched in β-sheet content [19], as well as toxicity from highly “purified” PrP^Sc^ and proteinase treated PrP^Sc^ extracted from the brains of terminally sick rodents [20, 21]. An *in vivo* model of acute neurotoxicity employing stereotaxic injection of recombinant full-length ovine PrP into the hippocampal CA2 region has been reported, with assessment for acute toxicity requiring morphological analysis approximately 24 hours later [22]. In addition, a model utilizing cultured organotypic cerebellar slice explants allowing assessment of factors that interfere with PrP^Sc^ replication and abrogate cerebellar granule cell loss has been described [23], although this model relies entirely on *de novo* PrP^Sc^ propagation to generate neurotoxic species over an extended 5–7 week period. This *ex vivo* culture model is arguably therefore not ideal for assessing direct acute PrP^Sc^ neurotoxicity because PrP^Sc^ propagation can closely correlate with deleterious cellular events such as heightened oxidative stress [24] that may also contribute to pathogenesis thereby potentially confounding the delineation of a directly neurotoxic PrP^Sc^ species. Of particular interest is a recent study demonstrating that PrP^Sc^ species cause retraction and subsequent loss of dendritic spines in cultured hippocampal neurons following several hours of exposure to PrP^Sc^ preparations [25]. This study however, reported that the synaptotoxicity required expression of PrP^C^ [25] leaving some uncertainty as to whether the neurotoxic PrP^Sc^ species were entirely those directly added to the culture or were different species generated through initial PrP^Sc^ propagation from host PrP^C^.

Electrophysiological studies employing techniques to assess LTP are an established method to explore potential acute neurotoxic effects of *ex vivo* brain material derived from neurodegenerative disorders such as Alzheimer’s disease (AD) [26]. Herein we report the use of an electrophysiology paradigm to explore the acute synaptotoxicity of *ex vivo* prion preparations derived from terminal disease brains briefly superfused onto hippocampal slices. We found that PrP^Sc^ (for convenience hereafter considered as synonymous with PrP^res^ species with at least modest PK resistance) is directly deleterious to LTP in the hippocampal CA1 region, with the degree of impairment approximating that observed during the natural evolution of prion disease in rodent models and independent of age of mice up to 11 months, with lysates from chronically M1000 prion infected cells (MoRK13-Inf) also inducing analogous acute synaptotoxicity. Additional detailed electrophysiological analyses suggested possible heightened pre-synaptic vulnerability to the acute synaptotoxicity, exemplified by impairment of post-tetanic potentiation (PTP) and correlating with failure of replenishment of the readily releasable pool (RRP) of vesicles during repeated high-frequency stimulation utilised for induction of LTP. Size fractionation chromatography demonstrated that synaptotoxic PrP correlated with PK-resistant species >100kDa, consistent with multimeric PrP^Sc^, with levels of these species >0.006 μg/ml appearing sufficient to induce synaptic dysfunction. Biochemical studies confirmed that synaptotoxic PrP^Sc^ in WT slices reduces essential proteins required for the induction and maintenance of hippocampal LTP such as pERK, pCREB, synaptophysin and vesicular glutamate transporter 1 (VGLUT1), as well as the NMDAR NR2A and NR2B subunits and the GluA2 subunit of AMPAR. Importantly, the PrP^Sc^ acute impairment of LTP and PTP was largely PrP^C^ independent, albeit with some noteworthy differences in the changes in key synaptic proteins and electrophysiological findings between wild type (WT) and *Prn-p* gene-ablated (PrPo/o) hippocampal slices, supporting the likelihood of non-PrP^C^ dependent mechanistic pathways. Dose-response assessments using cM1000 revealed similar sensitivity to synaptic disruption in PrPo/o and WT hippocampal slices. Our findings offer important new pathophysiological insights into the synaptic impairment underlying prion disease, enhancing prospects for development of targeted effective therapies.

## Materials and methods

### Ethics statement

All animal handling was in accordance with National Health and Medical Research Council (NHMRC) guidelines. All experimental procedures were approved by The Florey Institute of Neuroscience and Mental Health Animal Ethics Committee (Ethics number: 13–048) or the Biochemistry & Molecular Biology, Dental Science, Medicine (RMH), Microbiology & Immunology, and Surgery (RMH) Animal Ethics Committee, The University of Melbourne (Ethics number: 1312997.1).

### Animals

To prepare hippocampal slices for multi-electrode array (MEA) studies, 12-week-old and 11-month-old WT C57 black 6J (C57BL/6J) female mice were used (Animal Resource Centre, Western Australia), as well as 12-week-old female PrP knockout (PrPo/o) mice on a C57BL/6J background produced through 10 consecutive back-crossings of C57BL/6JX129/sv mice [27]. Mice were group caged, with 12-hour day-night light cycles and food and water provided *ad libitum*.

#### Brain homogenate and cell lysate preparation for electrophysiology studies

Whole brains from terminally ill mice inoculated with M1000 and MU02 prion strains [28, 29], as well as age-matched normal brain homogenate (NBH) “sham” inoculated mice, were homogenized to 20% (w/v) stocks in artificial cerebrospinal fluid (aCSF; 126mM NaCl, 2.5mM KCl, 26mM NaHCO_3_, 1.25mM NaH_2_PO_4_, 10mM Glucose, 1.3mM MgCl_2_.6H_2_O, 2.4mM CaCl_2_.2H_2_O) by passing through progressively smaller gauge needles (18g, 20g, 22g, 23g, 26g), sub-aliquoted and stored at -80°C until required. For each electrophysiology experiment, aliquots of 20% (w/v) prion-infected brain homogenate and NBH were diluted to a final concentration of 0.5% (w/v) in aCSF after pre-clearing at 100×g for one minute. These 0.5% (w/v) brain homogenates were the crude preparations (crude M1000: cM1000; crude MU02: cMU02; crude NBH: cNBH). Rabbit kidney epithelial (RK) cells expressing murine PrP^C^ (known as mouse RK13 or MoRK13; produced by Laura Vella and Andrew Hill as described in Vella et al. [30]), either mock-infected with NBH (control) or M1000 prion infected were cultured as described previously [31]. Control and M1000 infected cells were harvested, lysed in aCSF through needles as described above for brain homogenates to a final concentration 2% (w/v) for the use in electrophysiology experiments (control or mock-infected lysate, MoRK13-Un; M1000 infected lysate, MoRK13-Inf). In addition, we performed cM1000 serial dilution experiments on both WT and PrPo/o hippocampal slices to assess the sensitivity of the slices. For these experiments we utilised cM1000 brain homogenates (w/v in aCSF) diluted to 1%, 0.5%, 0.25% and 0.1%.

#### Proteinase K (PK) treatment of brain homogenate preparations for electrophysiological studies

M1000 brain homogenates and NBH, diluted to 0.5% (w/v) in aCSF and pre-cleared as above were treated with a final concentration of 5μg/mL PK for one hour at 37°C. The PK digested preparations (PK+M1000 and PK+NBH) were used immediately in electrophysiology experiments. Small aliquots of PK treated homogenates were stored at -80°C for subsequent biochemical analyses.

#### PrP immuno-depletion and PK-elution of PrP species from immuno-precipitated pellets of brain homogenates

In preparation for immuno-depletion, 50% protein-G-sepharose bead slurry (PGS; Invitrogen) was pre-blocked overnight at 4°C by incubation (with constant gentle movement) in 10% (w/v) skim milk powder in sterile phosphate buffered saline containing calcium and magnesium (PBS^Ca2+Mg2+^; Life Technologies). For each PrP immuno-depletion, 03R19 anti-PrP rabbit polyclonal antibody raised against residues 89–103 [29] was coupled to pre-blocked 50% PGS at room temperature (RT) for 2 hours. Normal rabbit serum (NRS) was utilized in the same manner as 03R19 to serve as a negative control. The 03R19 (or NRS) coupled PGS were incubated with 1% (w/v in aCSF) pre-cleared M1000 brain homogenate or NBH overnight at 4°C. PGS was pelleted by a pulse spin at 100 x g. Supernatants were collected as the PrP immuno-depleted samples and were further diluted 1:1 with aCSF to approximately a 0.5% (w/v) homogenate (PrP immuno-depleted NBH: dNBH; PrP immuno-depleted M1000: dM1000) prior to use in electrophysiology experiments. The immuno-captured PrP species bound to the pelleted PGS were resuspended in aCSF to the same starting volume of 1% (w/v) brain homogenate used for the immuno-depletion. PGS samples were then digested with a final concentration of 5μg/mL PK at 37°C for an hour with agitation (two cycles of 20 minutes agitation at 1400 rpm followed by 10 minutes with no agitation) to prevent the PGS from settling. The PGS was again pelleted (pulse spin at 100 x g) and the supernatant containing any eluted at least modestly PK-resistant PrP^Sc^ species was collected and diluted 1:1 with aCSF for use in electrophysiology experiments (PK eluted PrP immuno-precipitated NBH, PK+IP-NBH; PK eluted PrP immuno-precipitated M1000, PK+IP-M1000). Small aliquots of PK+IP-NBH and PK+IP-M1000 were retained and stored at -80°C for subsequent biochemical analyses.

### Hippocampal slice preparation for electrophysiology studies

Mouse brains were quickly collected following decapitation while under deep anaesthesia induced by isoflurane. 300μm dorsal horizontal brain slices were prepared using a vibratome (Leica VT1200S) in ice-cold continuously carboxygenated (5% CO_2_ and 95% O_2_) cutting solution (3mM KCl, 25mM NaHCO_3_, 1.25mM NaH_2_PO_4_, 206mM Sucrose, 10.6mM Glucose, 6 mM MgCl_2_.6H_2_O, 0.5mM CaCl_2_.2H_2_O). Approximately three optimal mid-hippocampal slices were collected from each hemisphere for electrophysiology studies. Slices were then allowed to stabilise at 32°C by incubation for one hour in continuously carboxygenated aCSF prior to mounting onto 60MEA200/30iR-Ti-pr-T multi-electrode arrays (MEA; Multichannel Systems; Germany) with secure placement achieved using Harp slice grids (ALA HSG-5B, Multichannel Systems; Germany) to ensure good contact of the CA1 region with the MEA (S1A(1) to S1A(3) Fig). Three slices were simultaneously mounted in separate recording chambers and were independently continuously superfused with carboxygenated aCSF (S1A(3) Fig panel i).

#### Electrophysiology paradigm

Hippocampal field excitatory post-synaptic potentials (fEPSP) were evoked by stimulating one of the MEA grid electrodes that was best aligned to the Schäffer collateral pathway while recording from other electrodes placed on the stratum radiatum of the CA1 region (S1A(3) Fig panel ii). The average number of electrodes recorded from and utilised for analysis in each slice was seven. The amplitude of fEPSP was recorded as the synaptic response. The basal stimulus intensity was determined by generating an input-output (I-O) curve with the intensity chosen sufficient to achieve a fEPSP of ~40% of the maximum response without causing a population spike and a baseline was recorded with stimulation every 30 secs for 30 mins. After approximately 10 mins of the 30-mins baseline, the hippocampal slice was then treated by superfusion with prion containing or control preparations for 5 mins, followed by the rest of the baseline recording in aCSF to ensure return of a stable baseline prior to trains of high frequency stimulation (HFS) (see S1A(4) Fig). The HFS trains (three 500 millisecond, 100Hz trains, 20 sec apart) were delivered (see S1A(4) Fig), followed by post-HFS stimulation every 30 sec for 30 mins, wherein the first response was the post-tetanic potentiation (PTP) and the responses recorded from five mins post-HFS considered the LTP. Immediately after the recordings, slices were snap-frozen and stored at -80°C for future biochemical analyses. For each independent experiment (n = 5–10 for each treatment), hippocampal slices generated from the same mouse brain were simultaneously utilized for perfusion with aCSF (technical control), NBH control preparations and prion infected samples.

Several synaptic neurophysiological parameters were recorded and analysed (see S1A(4) Fig):

1. I-O curve: fEPSP responses to a series of stimuli of increasing strength from 0 mV up to a stimulus that evoked the maximum fEPSP (ie 3000 mV-5000 mV determined by plateau responses), with the I-O curve prior to treatments (I-O 1) used to determine the basal stimulus intensity, as well as measure the strength of synaptic transmission before (I-O 1) and after treatments and LTP expression (I-O 2) (S1A(4) Fig, boxes 1 & 6).
2. PTP: The first fEPSP response at 0.5 sec after the third HFS train (see S1A(4) Fig, box 4; S1B Fig).
3. LTP: Responses recorded from five minutes following HFS trains represent LTP. The last 10 mins of LTP was used for analyses (S1A(4) Fig, box 5; S1B Fig).
4. Paired pulse facilitation (PPF) ratio can be used to estimate the basal probability of neurotransmitter release, which was measured by two identical basal stimuli delivered at a 20 millisecond interval with the fEPSP amplitude of the second stimulus divided by that of the first stimulus (see S1B Fig). PPF was measured before (PPF1) exposure to prion containing or NBH control preparations, as well as after 30 minutes of LTP expression (PPF2) (see S1A(4) Fig, box 1 and box 6).
5. Readily releasable pool (RRP) depletion: For each HFS train the first 9 evoked fEPSP pulses were utilised wherein the ratio of the pulse 1 and pulse 2 fEPSP amplitude (pulse 2 divided by pulse 1) estimates the probability of transmitter release (*Pr*) in each train (see S1C Fig). The fEPSP amplitude declined after pulse 2 to the last pulse in a HFS train and the rate of decline was used to estimate the RRP decays in each train (Further explained in Data analysis section) (see S1D Fig).
6. RRP replenishment: Relative changes in RRP size were estimated by extrapolating to the Y-intercept the best-fit straight line of the last 4 pulses (pulse 6 to pulse 9) of the cumulative fEPSP amplitudes of each HFS train [32–34] (Further explained in the Data analysis section) (see S1E Fig).
7. PPF time-course: For the time-course PPF study without LTP induction, the PPF1 was measured (with basal stimulation) before (PPF1A) and immediately after (PPF1B) exposure to either cM1000 or cNBH; following PPF1B, PPF was measured every 5 minutes for one hour. The slices were stimulated with basal stimulation during the treatment and at 5-minutely intervals (see S1F Fig).

Note: the omission of specific GABA_A_ receptor antagonists during measurements of fEPSP to calculate PPF ratios may reduce the accuracy of *Pr* estimates. Similarly, the omission of specific GABA_A_ receptor blockade during HFS may render estimates of the RRP based on changes in fEPSP amplitudes less accurate.

### Size exclusion chromatography

Prior to size exclusion chromatography, brain homogenates were solubilized in Sarkosyl (w/v in 1xPBS), dialysed and filtered. Normal brain homogenates (~10% w/v) were pelleted by 15000xg spin for 10 minutes at 4°C. The supernatant was discarded and the pellet was reconstituted with 4% (w/v in 1xPBS) Sarkosyl, incubated at 37°C for 30 minutes, and centrifuged at 10000xg for 10 minutes. The pellet was discarded, and the supernatant was collected and exhaustively dialysed (using 10kDa cut-off dialysis tubing) four times in 1x PBS dialysate (containing no Mg^2+^ or Ca^2+^) that was ~166 fold greater than the sample volume with each dialysis conducted overnight at 4°C. Parallel to these procedures, ~1% (w/v) PK+IP-M1000 was pelleted by 15000xg, and the pellet was resuspended in 4% (w/v) Sarkosyl with a volume that was 10-fold less than the initial volume to concentrate the PK+IP-M1000 into ~10% (w/v). Similar procedures were utilized to solubilize ~1% (w/v) dM1000 and concentrate to ~10% (w/v). The 10% (w/v) solubilized and dialysed preparations were filtered using a 0.22-micron filter before ~3mL was injected into a size exclusion chromatography column (HiPreP 16/60 s-100) at a flow rate of 0.5mL per min. The protein complexes were eluted in 1x PBS (containing Mg^2+^ and Ca^2+^) at 0.5mL per min flow rate, wherein the void volume was collected at ~70 minutes after injection followed by continuous collection of 1mL fractions every two minutes for 80 minutes (~40 fractions in total). The size of proteins or protein complexes fractionated by size exclusion chromatography and eluted into each fraction was determined following size fractionation of the following size exclusion chromatography markers: bovine erythrocyte carbonic anhydrase (~29kDa), bovine serum albumin (~66kDa), yeast alcohol dehydrogenase (~150kDa), sweet potato beta-amylase (~200kDa), horse spleen apoferritin (~443kDa), and bovine thyroglobulin (~669kDa). Fractions 1 (the void volume) through 12 were enriched for proteins, protein complexes or protein oligomers and protofibrils with molecular weight above ~100kDa, whereas fractions 15 through 30 were enriched for proteins with molecular weights less than ~100kDa, including monomeric proteins such as PrP^C^. The levels of prion proteins in each fraction were determined by western blotting, including before and after treatment with 5μg/mL PK for 60 minutes at 37°C.

### Biochemical analyses

Hippocampal slices (n = 5 for each treatment condition) were analysed after dissection of the hippocampus from surrounding tissue, homogenization in lysis buffer (50mM Tris-HCl pH 7.4, 150mM NaCl, 0.1% (w/v) SDS, 0.5% (w/v) sodium deoxycholate, 1% (v/v) NP-40) using needles as with whole brain homogenates, methanol precipitation of proteins by adding 5× volumes of ice-cold 100% methanol and incubating at -20°C overnight, followed by centrifugation at (20817xg) at 4°C for one hour. Supernatants were discarded and pellets were resuspended in 50μL lysis buffer and prepared in 4x sample buffer (NuPAGE LDS, Thermo Fisher Scientific) with a final concentration of 6% beta-mercaptoethanol. As required, aliquots of 1% brain homogenates (w/v in aCSF) of M1000, MU02 and NBH were also utilised for western blot analysis, including after digestion using 5 or 50μg/ml PK for 60 minutes at 37°C as indicated in the figure legends. The 5μg/ml PK digestion was used for all other PK treatments such as for the PrP-containing preparations used for hippocampal slice treatments (Fig 2D & 2G). In addition, for quantifying levels of PrP in brain homogenates and other preparations used in electrophysiology studies, a serial dilution of recombinant full-length mouse PrP (rPrP; made as described previously [35]) of known concentrations (prepared as described in [36]) was utilized to generate a standard curve of rPrP through probing by western blotting (using 8H4 anti-PrP antibody) and densitometric analysis, which was then used to estimate levels of PrP in the various preparations loaded onto the same gel. Proteins were analysed by PAGE and immunoblotting as described previously [37]. Briefly, samples were resolved on NuPAGE Novex 4–12% Bis-Tris gels (ThermoFisher Scientific), transferred to PVDF membrane (Millipore), blocked in either 5% (w/v) skim milk powder (SkM) or 3% (w/v) bovine serum albumin (BSA), probed with various antibodies (see S1 Table for a summary of primary and secondary antibodies utilized, their dilutions, as well as blocking conditions/antibody diluents), with protein detection using enhanced chemiluminescence (ECL Prime and Select, Invitrogen). Membranes were also stained with Coomassie blue (and de-stained) to determine relative total protein levels. All chemiluminescent and digital imaging was carried out using a Fujifilm LAS-3000 Intelligent dark box.

### Statistical analyses

Statistical analyses were performed using GraphPad Prism 6 (USA). The PTP and LTP fEPSP data were exported to Excel files (by LTP Analyzer software from Multichannel Systems) where they were normalized to average fEPSP recorded over the last five minutes of baseline recording. An unpaired Student t test (parametric test with Welch’s correction) was used to compare the average LTP and PTP of the treatment groups, such as NBH controls versus prion containing (or depleted) preparations. A paired Student t test (parametric test) was used to compare the average ratio of PPF1 and PPF2. I-O1 and I-O2 were compared using ANOVA with repeated measures. The fEPSP amplitudes of the HFS trains were quantified using PlotDigitizer software and normalized to the baseline fEPSP amplitude. The slope of decline of fEPSP amplitude from pulse 3 to the last pulse in each HFS train was compared between treatment groups by one phase decay exponential function in which the time constant of decay (Tau = 1/K) measures the rate of RRP decline in each train. The ratio between pulse 1 (P1) and pulse 2 (P2) of each train was compared between trains within a treatment group by paired Student t test to measure the probability of release per train. Cumulative fEPSP responses of each of the three trains were compared between treatment groups using a linear fit equation (of the last 4 cumulative fEPSPs/train) comparing Y-intercepts upon the initial stimulus after extrapolating the linear fit [32, 33]. Acute synaptotoxicity in the form of LTP and PTP change was calculated as the percentage decrease relative to their appropriate negative controls. The acute synaptotoxicity estimated in the form of PPF ratio was calculated as the percentage of PPF ratio decline in PPF2 relative to PPF1. Because the PPF ratio is inversely proportional to the *Pr*, the percentage of PPF ratio decline represents the *Pr* increase in PPF2. All data are presented as mean (m) ± standard error of mean (SEM). The western blot bands of interest were quantified by densitometry (Image J), after correcting for total protein level and analysed by Student unpaired t test (parametric test with Welch’s correction).

## Results

### M1000 and MU02 brain homogenates are acutely synaptotoxic to mouse hippocampal CA1 region

**Fig 1:**
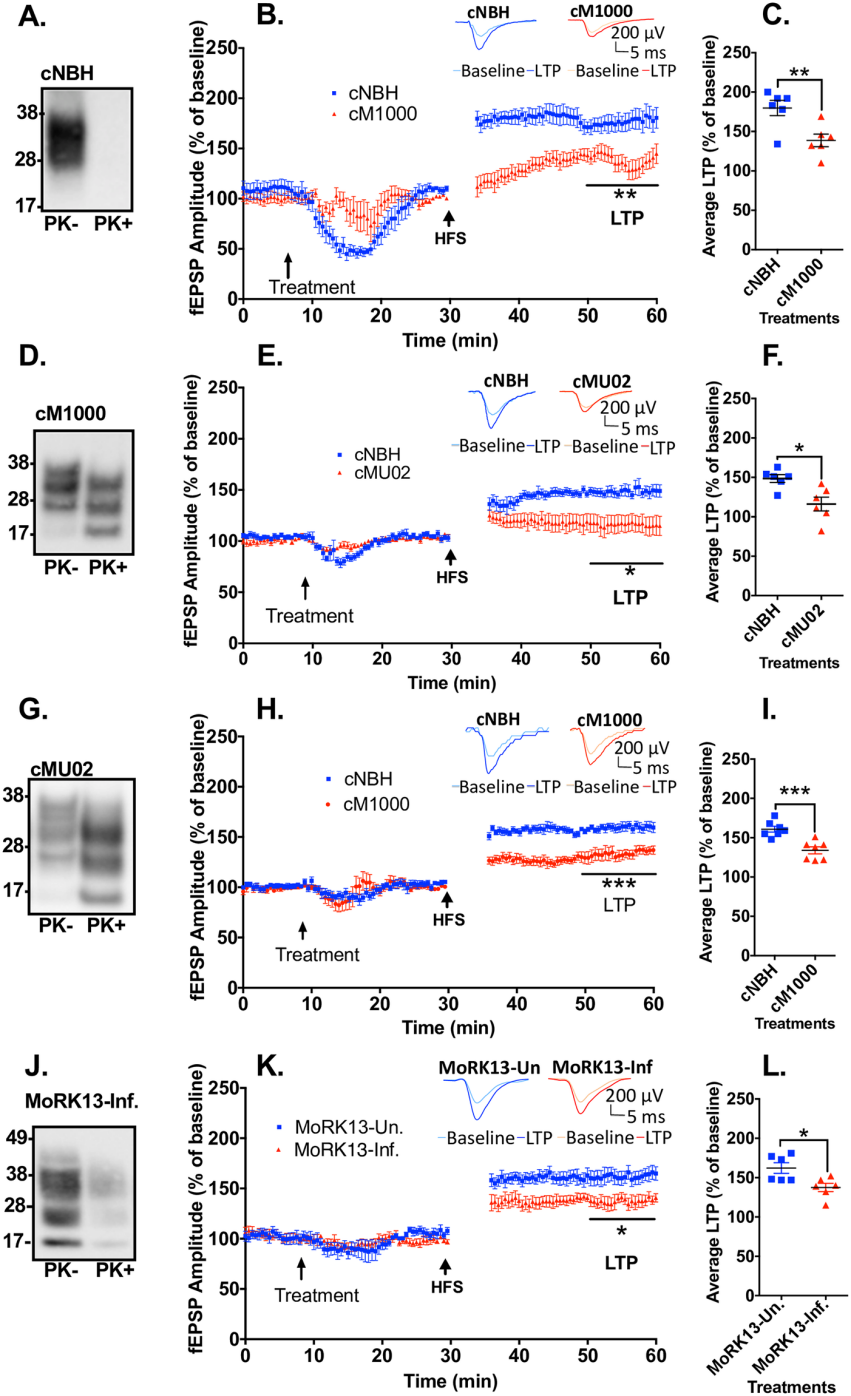
Crude ex vivo PrP^Sc^ containing preparations acutely impair LTP. (A) Western blot of cNBH not treated (-) or treated (+) with 50μg/ml PK. The PK- cNBH was utilised as control preparations for the treatments of WT hippocampal slices using prion-containing crude brain homogenates. (B) cM1000 superfused for 5 minutes over hippocampal slices from 12-week old WT mice approximately 20 minutes prior to HFS caused a significant impairment of LTP with (C) average LTP reduced by 53 ± 9% (n = 6) compared to cNBH. (*p = 0*.*0087*). (D) Western blot of cM1000 not treated (-) or treated (+) with 50μg/ml PK with PK+ proving presence of PrP^Sc^. (E) cMU02 superfused for 5 minutes over slices from 12-week old WT mice approximately 20 minutes prior to HFS caused a significant impairment of LTP with (F) average LTP reduced by 62 ± 19% (n = 6) compared to cNBH. (*p = 0*.*0129*). (G) Western blot of cMU02 not treated (-) or treated (+) with 50μg/ml with PK+ proving presence of PrP^Sc^. (H) The PK- cM1000 was superfused for 5 minutes over slices from 11-month old WT mice approximately 20 minutes prior to HFS caused significant impairment of LTP with (I) average LTP reduced by 44 ± 7%; (n = 7) compared to cNBH. (*p = 0*.*0006*). (J) Western blot of MoRK13-Inf not treated (-) or treated (+) with 50μg/ml PK with PK+ proving presence of PrP^Sc^. (K) The PK- MoRK13-Inf was superfused for 5 minutes over slices from 11-month old WT mice approximately 20 minutes prior to HFS caused significant impairment of LTP with (L) average LTP reduced by 40 ± 6% (n = 6) compared to MoRK13-Un (*p = 0*.*0172*). (B, E, H & K) The first five-minute fEPSP recordings following HFS trains have been removed to enhance clarity and the last 10 minutes of post-HFS recordings were used for LTP analysis. (A, D, G, J) Molecular markers are provided at left. (B, E, H, K) Examples of raw fEPSP traces are provided as insets. Data are presented as ± SEM. *p<0.*05*, **p<0.*01*, ***p<0.*001*, ****p<0.*0001*.

Brains of terminally sick prion infected mice are presumed to contain all pathogenic species responsible for the development of prion diseases. To determine if some of these species are acutely synaptotoxic, independent of *de novo* propagation of PrP^Sc^ given the very short time-frame of the experiments, crude brain homogenates were introduced onto *ex vivo* mouse hippocampal slices to determine any deleterious effects on LTP. These crude homogenates were derived from WT C57BL/6J mice intracerebrally inoculated with normal brain homogenate (cNBH) and terminally ill mice infected with either of two mouse-adapted human prion strains, M1000 (cM1000) [28] and MU02 (cMU02) [29]; Fig 1A, 1D and 1G provide examples of PrP^res^ detection by western blots of brain homogenates pre- and post-PK treatment. The hippocampal CA1 region LTP of 12-week-old WT mice was significantly reduced by 53 ± 9% (n = 6) following five-minute exposure to cM1000 (Fig 1B & 1C; see S2A Fig) and by 62 ± 19% (n = 6) following exposure to cMU02 (Fig 1E & 1F; see S2A Fig) relative to cNBH. There was no significant difference between the acute synaptotoxicity of cM1000 and cMU02 (see S2A Fig). Further, there was no significant difference in the degree of LTP disruption of cM1000 in slices generated from 11-month-old WT mice (impaired by 44 ± 7%; n = 7) compared with 12-week-old WT mice (Fig 1H & 1I; see S2A Fig). Consistent with the LTP impairment, the I-O2 curve was not significantly enhanced compared to the I-O1 curve following exposure to cM1000 (in both 12-week-old WT and 11-month-old WT mice) and cMU02 compared with cNBH (see S2E and S2F Fig). In addition, relative to aCSF technical controls, cNBH negative controls did not affect LTP (S2B & S2C Fig).

### Lysates from M1000-infected MoRK13 cells are acutely synaptotoxic

Propagation of *bona fide* prions in MoRK13 cell lines that express murine PrP^C^ has been well established through studying M1000 and MU02 *ex vivo* transmission [29, 31]. PK-resistant PrP^Sc^ detected in these cells (Fig 1J) appears a valid biomarker for successful transmission of prions. To determine if these cells also propagate acutely synaptotoxic species similar to cM1000, whole cell lysates derived from MoRK13-Inf were also briefly superfused onto hippocampal slices from 11-month-old WT mice, with the amount of biochemically detectable PrP^Sc^ in MoRK13-Inf lysates balanced to equate that in cM1000. Similar concentration MoRK13-Un lysates did not affect LTP relative to aCSF controls, demonstrating no background toxicity of the uninfected cell lysates (see S2D Fig). Following brief treatment with MoRK13-Inf lysates, the LTP was significantly impaired by 40 ± 6% (n = 6) (Fig 1K & 1L), with the degree of LTP disruption similar to that obtained from cM1000 (see S2A Fig). Consistent with the LTP impairment, the I-O2 also failed to significantly increase relative to I-O1 following exposure to MoRK13-Inf compared with MoRK13-Un (see S2H Fig).

### Acute synaptotoxicity of cM1000 is directly associated with PrP species

**Fig 2:**
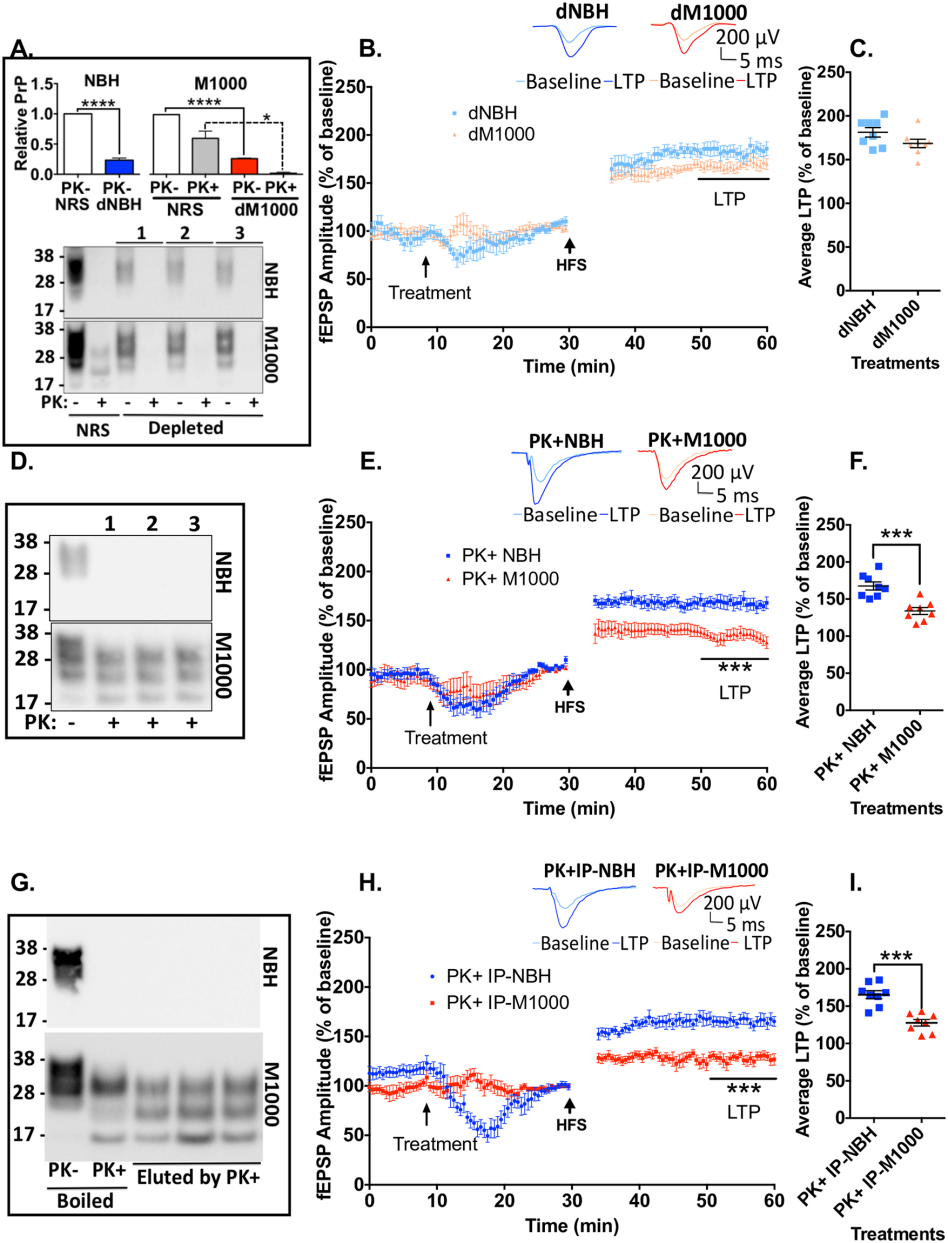
PK-resistant PrP species are responsible for acute synaptotoxicity. (A) PrP immuno-depletion selectively depleted ~77 ± 12% of PrP^C^ (dNBH) from cNBH and ~77 ± 9% total PrP species and 96 ± 4% of PK-resistant PrP (dM1000) from cM1000 relative to the normal rabbit serum (NRS) controls. Controls and depleted preparations were either not treated (-) or treated (+) with 50μg/ml PK to determine the level of PK-resistant PrP. (B) dM1000 superfused for 5 minutes over hippocampal slices from 12-week old WT mice approximately 20 minutes prior to HFS trains displayed significant rescue of LTP with (C) average LTP increased 74 ± 14% (n = 8), which was no longer significantly different to slices superfused with dNBH. (D) Western blot of cNBH and cM1000 treated with 5μg/mL PK (PK+NBH; PK+M1000), which was sufficient to completely degrade all PrP^C^ in cNBH. (E) PK+M1000 superfused for 5 minutes over slices from 12-week old WT mice approximately 20 minutes prior to HFS significantly impaired LTP with (F) average LTP reduced by 48 ± 7% (n = 8) relative to PK+NBH preparations (*p = 0*.*003*). (G) Pellets generated through PrP immuno-precipitation (IP) of total PrP from cNBH and cM1000 were resuspended and western blotted after 5μg/mL PK digestion to specifically elute at least modestly PK resistant PrP^Sc^, with comparison made to NBH and M1000 resuspended pellets wherein PK-resistant PrP was eluted by boiling after PK digestion with 5 μg/ml PK. (H) The resuspended, PK-eluted IP pellets from cNBH (PK+IP-NBH) or cM1000 (PK+IP-M1000) superfused for 5 minutes over slices from 12-week old WT mice approximately 20 minutes prior to HFS caused significant impairment of LTP measured during the last 10-minutes of post-HFS recording, with (I) average LTP reduced by 59 ± 5% (n = 8) relative to PK+IP-NBH preparations (*p = 0*.*001*). (B, E & H) The first five-minute fEPSP recordings following HFS trains have been removed to enhance clarity. (A: lower panel, D, G) Molecular markers are provided at left. (B, E, H) Examples of raw fEPSP traces are provided as insets. Data are presented as ± SEM. *p<0.*05*, **p<0.*01*, ***p<0.*001*, ****p<0.*0001*.

PrP^Sc^ species are readily detectable in the brains of terminal prion infected mice [38] and PrP^Sc^ species closely correlate with the disruption of neuronal structures including dendritic spines in prion disease *in vivo* mouse models [16] and a primary neuronal cell culture model [25]. To determine the relationship of PrP species to the acute disruption of LTP following exposure to cM1000 in our electrophysiology assay, total PrP species were selectively immuno-depleted from both cM1000 and cNBH under native conditions using the 03R19 antibody [29] coupled to protein-G conjugated sepharose beads. As shown in Fig 2A, relative to the normal rabbit serum (NRS) control, the 03R19 immuno-depletion selectively reduced ~77 ± 12% (n = 9) of PrP^C^ from cNBH (dNBH) and ~77 ± 9% total PrP species and 96 ± 4% PrP^res^ from cM1000 (dM1000). The immuno-depletion did not introduce any synaptotoxicity, wherein dNBH did not affect LTP relative to aCSF controls (see S3B Fig). When hippocampal slices generated from 12-week-old WT mice were treated with dM1000, LTP was not significantly different to dNBH, and was therefore effectively ‘rescued’ by 74 ± 14% (n = 8) when compared to cM1000 (Fig 2B & 2C), clearly supporting that PrP species in cM1000 are directly responsible for the LTP disruption. In addition, I-O2 became significantly increased relative to I-O1 following exposure to the dM1000 compared with dNBH (see S3G Fig), thus verifying substantial rescue of the synaptic transmission concomitant with the recovery of LTP.

### At least modestly PK-resistant PrP^Sc^ species in cM1000 are responsible for acute synaptotoxicity

Both PK-sensitive and PK-resistant species have been found capable of transmitting prion disease [39]. To determine whether any PK-resistant PrP^Sc^ species were directly responsible for the LTP disruption observed with cM1000, cM1000 was treated with PK prior to superfusion over WT hippocampal slices. The mild PK treatment (5μg/ml for one hour at 37°C) digested ~90% of total protein from cM1000 relative to before the PK treatment but importantly a prominent amount of PK-resistant PrP^Sc^ was still evident on western blotting after the PK digestion, demonstrating that the modest PK treatment had considerably enriched for PrP^Sc^ (Fig 2D lower panel; see S3H Fig columns i-ii & S3I Fig). The mild PK treatment digested ~80% of total proteins from cNBH including all PrP^C^ relative to before the PK treatment (Fig 2D upper panel; see S3H Fig columns v-vi & S3I Fig). Importantly, this mild PK treatment and possible by-products did not cause any background synaptic impairment when comparing PK+NBH relative to the aCSF technical control (see S3C Fig). Hippocampi derived from 12-week-old WT mice exposed to PK+M1000 demonstrated significant LTP impairment of 48 ± 7% (n = 8) relative to PK+NBH (Fig 2E & 2F). Thus, the LTP disruption of the PK+M1000 was approximately equivalent to that of the cM1000 (see S2A & S3A Figs), indicating that at least modestly PK-resistant PrP^Sc^ species are most likely directly responsible for the acute synaptic disruptions caused by cM1000. This dysfunction correlated with the failure of I-O2 to significantly increase relative to I-O1 after treatment with PK+M1000 (see S3E Fig).

To further validate that PrP^Sc^ species, in particular PK resistant species, are directly responsible for the acute synaptotoxicity, the total PrP species immuno-precipitated (IP) from cM1000 (IP-M1000) and cNBH (IP-NBH) (using the method employed for PrP immuno-depletion) were eluted from the IP pellets by the same modest PK treatment (5μg/ml for one hour at 37°C). This PK elution digested ~70% of total proteins from IP-NBH pellets including all PrP^C^ (Fig 2G upper panel; see S3H Fig columns vii-viii, & S3I Fig) and ~80% of total proteins from IP-M1000 pellets, leaving substantial levels of PrP^Sc^ in the preparations as revealed by western blotting (Fig 2G lower panel; see S3H Fig columns iii-iv, & S3I Fig), effectively enriching the preparations for modestly PK-resistant PrP^Sc^. Following brief exposure of 12-week-old WT hippocampal slices to the PK-eluted IP-M1000 (PK+IP-M1000), LTP was significantly impaired by 59 ± 5% (n = 8; Fig 2H & 2I). Noteworthy is that the degree of LTP disruption caused by the PrP^Sc^ alone from IP pellets was at least as great as that obtained from cM1000 and PK+M1000 (see S2A & S3A Figs), strongly supporting that these species were responsible for the LTP disruption observed with other M1000 preparations. Consistent with the LTP dysfunction, the I-O2 was also prevented by PK+IP-M1000 from becoming significantly enhanced after LTP induction relative to I-O1 (see S3F Fig).

### Multimeric PrP^Sc^ species present in M1000 preparations correlate with the acute synapototoxicity to LTP

**Fig 3:**
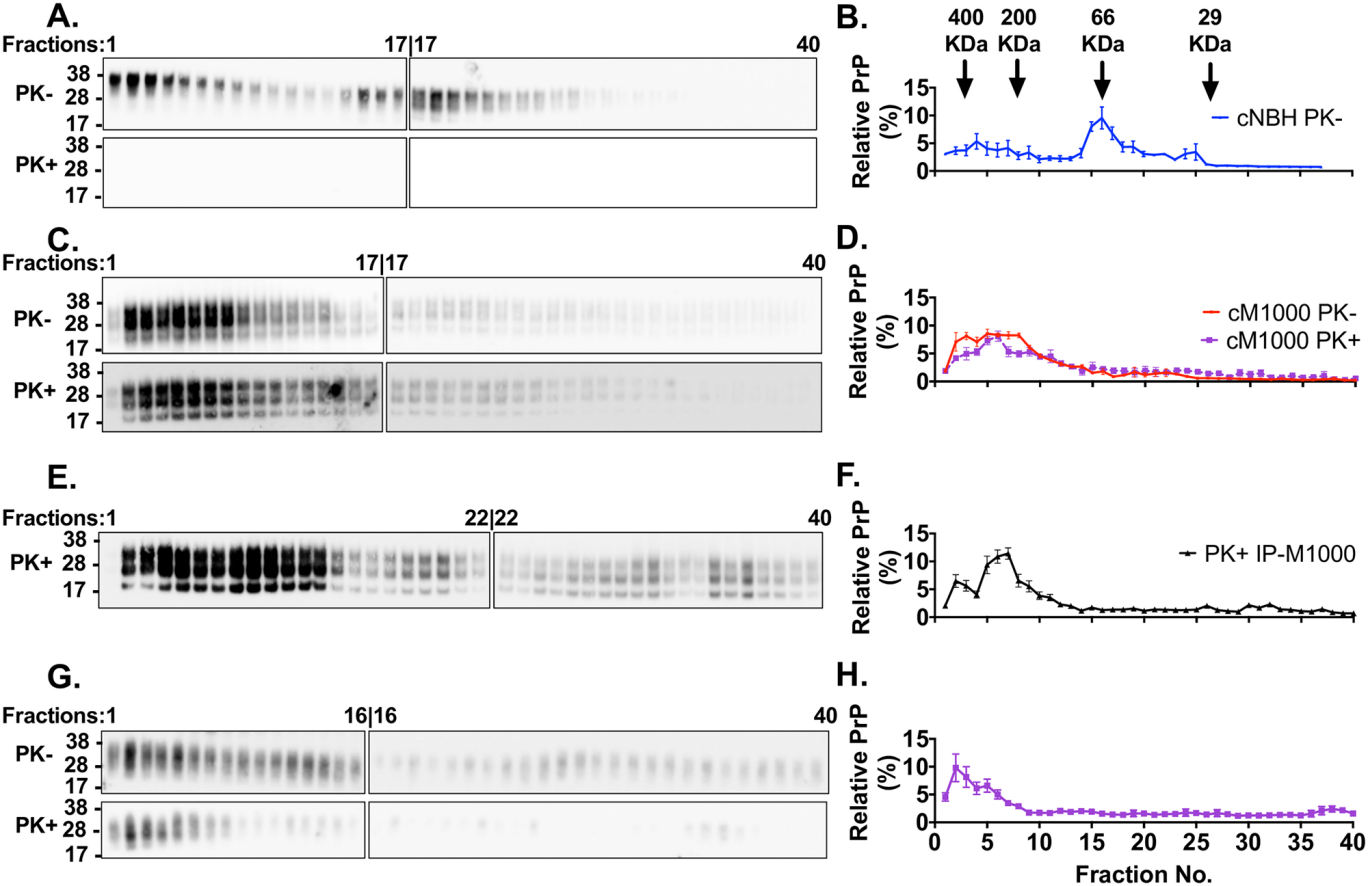
Prion acute synaptotoxicity is associated with increased levels of multimeric PrP^Sc^ species in *ex vivo* M1000 preparations. (A) Representative anti-PrP immunoblots of fractions collected following size exclusion chromatography of 10% (w/v) cNBH after clarifying centrifugation, solubilization in 4% (w/v) Sarkosyl and exhaustive dialysis. The fractions were immunoblotted before (-) and after (+) mild PK treatment (PK; 5μg/mL PK; incubated at 37°C for an hour). (B) Densitometric analysis of the immunoblots of cNBH fractions (n = 3) displayed as the percentage of PrP in each fraction over the sum of PrP levels in all the fractions. (C) Representative anti-PrP immunoblots of fractions collected following size exclusion chromatography of 10% (w/v) solubilized, dialyzed, and pre-cleared cM1000. The fractions were immunoblotted before (-) and after (+) mild PK treatment as described for (A). (D) Densitometric analysis of the immunoblots of cM1000 fractions (n = 3). (E) Representative anti-PrP immunoblots of fractions collected following size exclusion chromatography of at least modestly PK-resistant PrP^Sc^, extracted from 1% (w/v) cM1000 by immunoprecipitation (IP) and eluted by a mild PK digestion (5μg/mL PK at 37°C for an hour) before being concentrated (10-fold to be equivalent to 10% w/v), solubilized, dialyzed, and pre-cleared for the size fractionation (PK+IP-M1000). (F) Densitometric analysis of the immunoblots of PK+IP-M1000 fractions (n = 2). (G) Representative anti-PrP immunoblots of fractions collected following size exclusion chromatography of solubilized, dialyzed, and pre-cleared 10% (w/v) M1000 brain homogenates containing only ~30% of the total PrP following selective removal of ~70% by immuno-depletion (dM1000). The fractions were immunoblotted before (-) and after (+) mild PK treatment (5μg/mL PK at 37°C for an hour). (H) Densitometric analysis of the immunoblots of dM1000 fractions (n = 3). (D & H) The levels of fractionated PrP with and without PK treatment were overlaid for illustration purposes and cannot be compared quantitatively because they were analysed independently. The effective molecular weights of PrP species collected in fractions one through 12 were larger than 100kDa consistent with multimeric species while those collected in fractions 15 through 40 were smaller than 100kDa (Molecular weight markers are indicated as arrows above B. (A, C, E, and G) Since all 40 fractions could not be loaded onto one 26-well gel, two 26-well gels were used for immunoblotting of the fractions from one size exclusion chromatography experiment; importantly the last fraction on the first gel was also loaded as the first on the second gel, thereby allowing the levels of PrP on the second blot to be normalized to those on the first blot. The PrP species were probed by either anti-PrP 03R19 (in A, C, and G) and 8H4 (in E) antibody. (A, C, E, and G) Molecular weight markers are provided at left. (B, D, F, and H) The data are presented as mean ± SEM.

Similar to misfolded pathogenic proteins responsible for other neurodegenerative diseases such as amyloid-beta (Aβ) in AD and alpha-synuclein in Parkinson’s disease, the neurotoxic species in prion diseases is believed to be soluble multimers or oligomers [40, 41]. Considerable data supports that PrP^Sc^ species accumulate into different size multimers such as oligomers and protofibrils, correlating with the natural evolution of prion disease and the onset of clinical signs in mice [42, 43]. To determine any correlation between the presence of multimeric PrP^Sc^ species in M1000 preparations causing acute synaptic dysfunction (especially cM1000, and PK+IP-M1000), PrP^Sc^ species in these preparations underwent size exclusion chromatography and analysis by western blotting. For the negative control, PrP^C^ species in cNBH were also fractionated by size exclusion chromatography. Through the use of size exclusion markers with molecular weights of ~400kDa, ~200kDa, ~66kDa and ~29kDa, protein complexes larger than 100kDa, including PrP^Sc^ multimers, were eluted in fractions 1 through 12, whereas protein species smaller than 100kDa, including PrP^C^, PrP^Sc^ monomers and endoproteolytic fragments were eluted in fractions 14 through 40. Relative to fractions of cNBH where most PrP^C^ species were eluted as monomers (Fig 3A and 3B), the fractions of cM1000 contained mostly multimeric PrP^Sc^ species (Fig 3C upper panel and Fig 3D) wherein significant levels were at least modestly PK-resistant (Fig 3C lower panel and Fig 3D). Interestingly, fractions of PK+IP-M1000 also contained predominantly at least modestly PK-resistant PrP^Sc^ multimers. In contrast, fractions of dM1000 contained significantly reduced levels of the multimeric PrP^Sc^ species, especially fractions 5–10, including at least modestly PK-resistant multimeric PrP^Sc^ species (Fig 3G and 3H), which were present at substantial levels in cM1000 (Fig 3C & 3D) and PK+IP-M1000 (Fig 3E & 3F). Consequently, the increased levels of multimeric PrP^Sc^ species in fractions 5–10 of cM1000 and PK+IP-M1000 which were relatively depleted in dM1000 appear most strongly correlated with the acute impairment of LTP (Fig 2).

### Detailed electrophysiological analyses suggests acute synaptotoxicity of PrP^Sc^ may be associated with heightened pre-synaptic vulnerability

#### PrP^Sc^ appears to reduce the probability of release (*Pr*) following expression of disrupted LTP

**Fig 4:**
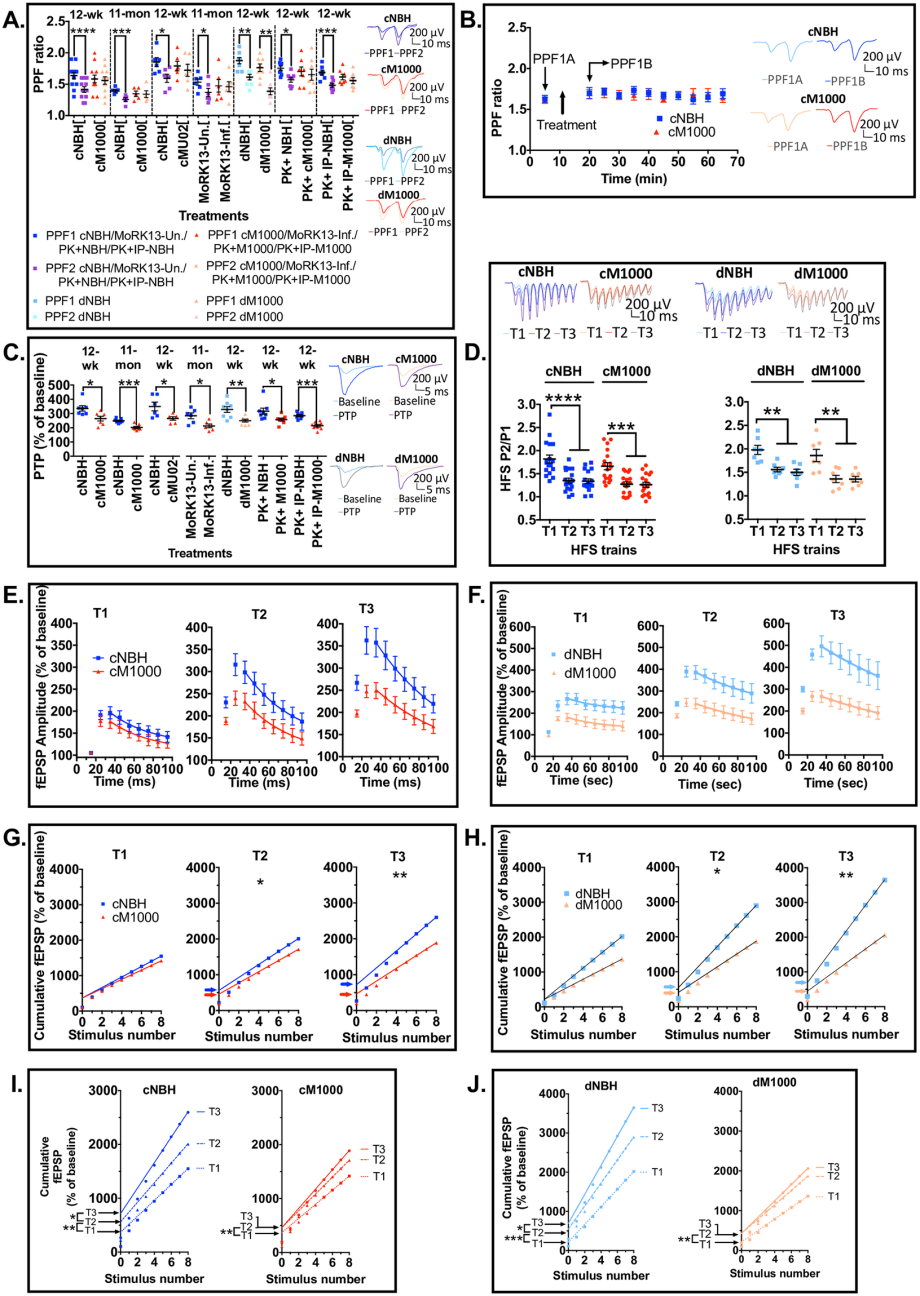
Prion acute synaptotoxicity demonstrates enhanced pre-synaptic vulnerability in WT hippocampal slices. (A) Normal PPF ratio reductions (the ratio becomes significantly reduced in PPF2 relative to PPF1) were obtained in slices after LTP induction and exposure to all negative controls: cNBH (for cM1000 treatments in 12-week old [*p<0001*] and 11-month old mice [*p = 0*.*0008*]; cMU02 treatment [*p = 0*.*0104*]); dNBH (*p = 0*.*0017)*; PK+NBH (*p = 0*.*0182*); PK+IP-NBH (*p = 0*.*0002*); and MoRK13-Un (*p = 0*.*0170*). Conversely, PPF ratio in slices treated with cM1000 (both in 12-week and 11-month old mice), cMU02, MoRK13-Inf, PK+M1000, and PK+IP-M1000 was not reduced in PPF2 relative to PPF1, showing poor *Pr* associated with the LTP disruption. The PPF ratio was normal in dM1000 (*p = 0*.*0013*) concomitant with the recovery of LTP. (B) Treatment of slices with cM1000 without inducing LTP expression did not affect PPF ratios relative to slices treated with cNBH. (C) Relative to appropriate negative controls, PTP was significantly disrupted after cM1000 treatment of slices from 12-week-old (*p = 0*.*0144*) and 11-month-old (*p = 0*.*0006*) mice. Similar PTP disruption was obtained following treatment with cMU02 (*p = 0*.*0391*), MoRK13-Inf (*p = 0*.*0172*), dM1000 (*p* = 0.0062), PK+M1000 (*p = 0*.*0359*), and PK+IP-M1000 (*p<0*.*0001*). (D) The *Pr* during the HFS trains was normal in both cM1000 and dM1000 relative to cNBH and dNBH controls, respectively where the *Pr* became significantly increased (determined by the P1 to P2 ratio in each train) in T2 and T3 relative to T1. (E & F) Consistent with the normal *Pr*, the rate of RRP depletion was normal across three HFS trains in both cM1000 (E) and dM1000 (F), where the time constant of decay between P3 and P9 in each train was not different between cNBH and cM1000, as well as between dNBH and dM1000. (G & H) The size of RRP (determined by the Y-intercepts of the linear fit of the last four pulses of HFS) became significantly diminished at T3 in cM1000 (G) and dM1000 (H) relative to cNBH (G) and dNBH (H). (I & J) This reduction in RRP size was caused by a significant impairment of the RRP replenishment (indicated by the increase in the RRP size between trains) to refill the RRP at T3 in both cM1000 (I) and dM1000 (J) compared with cNBH (I) and dNBH (J). The reduction in RRP size contributed directly to the impairment of PTP. (A-D) Examples of raw fEPSP traces are provided as insets. Data are presented as ± SEM. *p<0.*05*, **p<0.*01*, ***p<0.*001*, ****p<0.*0001*.

The paired pulse facilitation (PPF) ratio was used to determine if the *Pr* from docked neurotransmitter vesicles was disrupted and thereby contributing to the impairment of LTP in WT hippocampal slices. The PPF ratio is inversely related to the *Pr*, becoming significantly reduced following LTP (PPF2) relative to before LTP induction (PPF1) due to an increase in the *Pr*. A normal reduction in the PPF2 ratio was demonstrated in all negative controls including cNBH similar to that obtained in aCSF technical controls (Fig 4A; see S4A & S4B Fig). Conversely, the PPF2 ratio did not significantly reduce after exposure to any prion containing preparations (cM1000, MoRK13-Inf, cMU02 and PK+IP-M1000) (Fig 4A; see S4B Fig). These data suggest there may be a significant failure of *Pr* to increase during LTP induction, thereby contributing to and correlating with the impairment of LTP. There was no difference in the degree of *Pr* impairment obtained in 12-week-old WT mice compared with 11-month-old WT mice (see S4B Fig). Importantly, the PPF ratio decline returned when slices were superfused with using dM1000, correlating with the rescued LTP (Fig 4A; see S4B Fig).

Because the impairment of PPF ratio decline in prion containing preparations had only been measured after high frequency stimulation (HFS) trains utilised to induce LTP, it remained unclear whether the PPF ratio became disrupted immediately following exposure to PrP^Sc^. To determine if PrP^Sc^ may directly impair PPF independent of LTP expression, PPF was measured before (PPF1A) and shortly after (PPF1B) exposure to cM1000 without HFS. Baseline recordings were temporarily disrupted during the five-minute exposure to cNBH and cM1000 (maximal at ~15 minutes of recording) but quickly recovered to normal baseline levels (S4E Fig). Interestingly, the PPF ratio after exposure to cM1000 did not differ from cNBH, nor did the PPF ratio decline over the next ~ 50 minutes (Fig 4B; n = 8), suggesting that PrP^Sc^ does not appear to directly impair *Pr* and that the mechanisms underlying disruption of *Pr* by PrP^Sc^ are only manifested in the context of induction and expression of LTP.

#### PrP^Sc^ appears to disrupt post-tetanic potentiation in WT hippocampal slices by impairing the action potential dependent replenishment of the readily releasable pool (RRP) of vesicles during HFS trains, while *Pr* and mechanisms of neurotransmitter release remain normal

In addition to the PPF ratio, PTP was another pre-synaptic parameter assessed immediately after HFS trains. Relative to appropriate negative controls where PTP was not affected compared with aCSF controls (see S4C Fig), cM1000, cMU02, and MoRK13-Inf significantly disrupted PTP, further supporting that pre-synaptic impairment may be a key component of the acute synaptotoxicity, which was evinced by HFS (Fig 4C). Similar to the LTP impairment, the PTP disruption was age independent up to 11 months old (see S4D Fig). Further, PTP disruption was still evident when using PK+M1000 and PK+IP-M1000 (Fig 4C; S4D Fig), supporting that this PTP disruption also appears directly associated with modestly PK-resistant PrPSc. Noteworthy, however, in contrast to the significant rescue of both LTP and PPF, the PTP remained significantly impaired when exposed to dM1000 (Fig 4C; see S4D Fig), thereby further suggesting a possible enhanced pre-synaptic vulnerability to the acute synaptotoxicity possibly linked to the residual PrPSc, including some multimeric species (Fig 3G & 3H) in these preparations. PTP is strongly influenced by the *Pr*, RRP size, RRP depletion rate, and RRP replenishment rate, which are all action potential-elicited Ca2+-dependent functions in the pre-synaptic terminal during HFS trains [44]. Detailed electrophysiological analyses showed the *Pr* (Fig 4D) and RRP depletion rate (Fig 4E & 4F) appeared normal across the three HFS trains in both cM1000 and dM1000 relative to cNBH and dNBH, respectively demonstrating that mechanisms of neurotransmitter release are probably not impaired in PTP disruption; however, the size of the RRP, while normal in train 1 (T1) became progressively and significantly diminished in trains 2 (T2) and 3 (T3) in both cM1000 (n = 17) and dM1000 (n = 7) (Fig 4G & 4H) as a result of significant disruption of RRP replenishment in T2 and T3 (Fig 4I & 4J), thereby probably contributing to the disruption of PTP after exposure to both M1000 preparations.

#### PrP^Sc^ acute synaptotoxicity in WT hippocampi is associated with decreased expression levels of pre- and post-synaptic components crucial for LTP induction and maintenance

**Fig 5:**
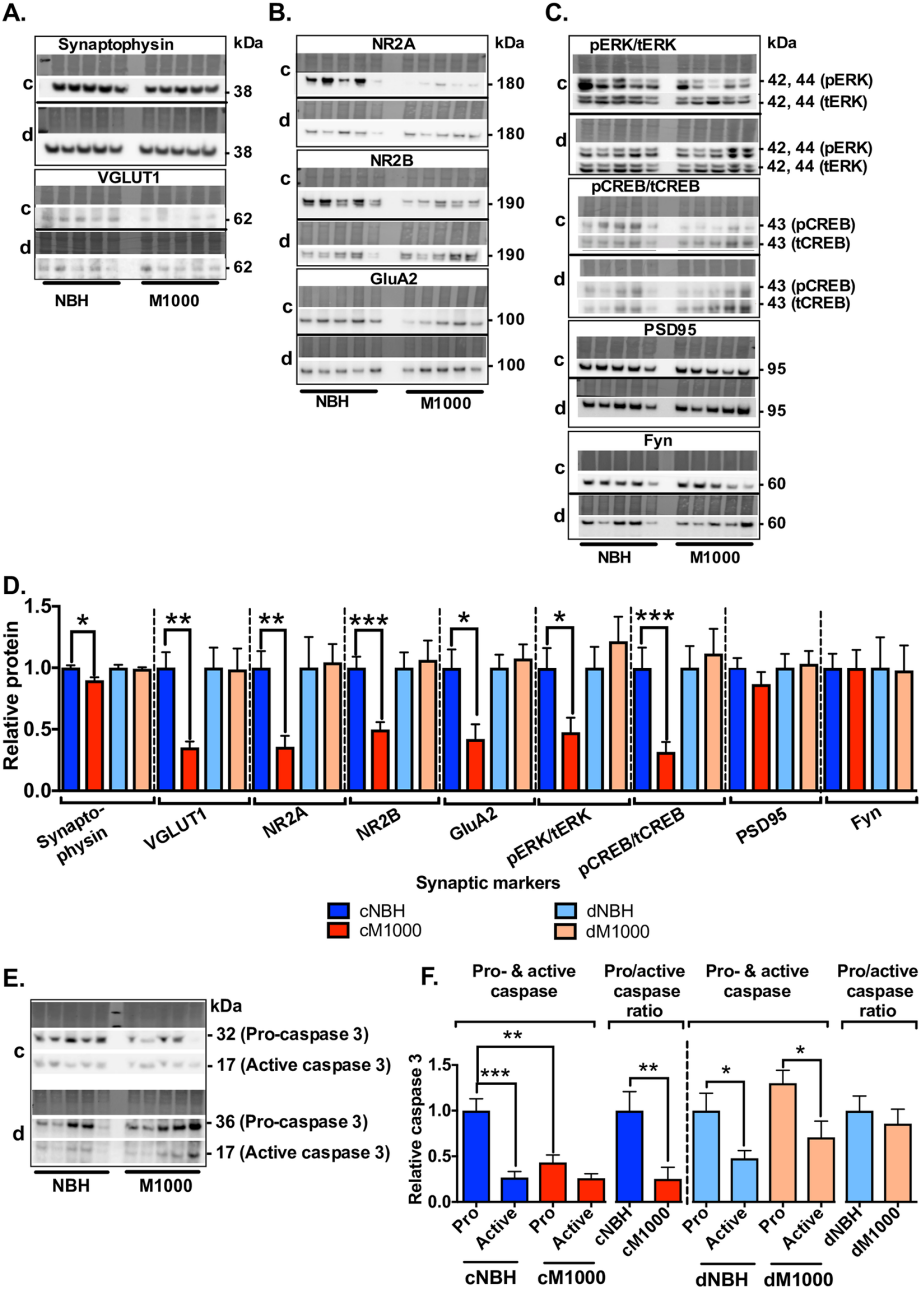
Altered expression levels of key pre- and post-synaptic markers correlate with synaptic dysfunction in WT hippocampi. Following induction of LTP, hippocampi were homogenized and biochemically analysed for key synaptic markers. (A & D) Hippocampi treated with cM1000 [*c*] expressed reduced levels of pre-synaptic markers, synaptophysin (*p = 0*.*0280*) and VGLUT1 (*p = 0*.*0108*), relative to those treated with cNBH [*c*]. Conversely, WT hippocampi treated with dM1000 [*d*] were associated with unaltered synaptophysin and VGLUT1 levels relative dNBH [*d*]. (B & D) NR2A/B-containing NMDAR were also reduced in hippocampi treated with cM1000 (NR2A: *p = 0*.*0012*; NR2B: *p = 0*.*0003*) relative to those treated with cNBH, with unaltered levels when employing dM1000 and dNBH. GluA2-containing AMPAR were also reduced in hippocampi treated with cM1000 relative to those treated with cNBH (*p = 0*.*0077)*, with unaltered levels when employing dM1000 and dNBH. (C & D) Expression levels of pERK and pCREB were reduced in slices exposed to cM1000 (pERK: *p = 0*.*0339*; pCREB: *p = 0*.*0108*) relative to cNBH but were unaltered when employing dM1000 and dNBH. PSD95 remained unaltered after exposure to both cM1000 and dM1000 supporting no loss of pre-existing synapses in prion acute synaptotoxicity. Fyn levels remained unaffected after treatment with either cM1000 or dM1000. (E & F) Relative to cNBH, cM1000 reduced expression levels of procaspase 3 (*p = 0*.*0028*) with unaltered production of the active form, thereby significantly reducing the pro- to active caspase 3 ratio (*p = 0*.*0083*). The pro-caspase 3 expression level was unaltered after treatment with dM1000 compared with dNBH with a normal pro- to active caspase 3 ratio. (A-C, E) Molecular weight markers are provided at right. Data are presented as ± SEM. *p<0.*05*, **p<0.*01*, ***p<0.*001*, ****p<0.*0001*.

Altered expression levels of core synaptic components integral for maintaining normal synaptic functions, have been frequently reported as features of Aβ42 toxicity [45]. The disruption we observed, especially the PPF ratio and PTP disruption, suggested the likelihood of perturbed expression of key pre-synaptic markers. Western blotting of WT hippocampi with impaired PPF2 ratios revealed a significant down-regulation of the synaptic vesicle proteins synaptophysin and VGLUT1, relative to hippocampi with normal PPF2 ratios (Fig 5A & 5D). It is important, however to acknowledge that because PTP reflects short-term synaptic potentiation, which lasts only a few minutes after HFS trains, and the tissues were harvested following LTP expression, these altered pre-synaptic markers represent the pre-synaptic concomitants of LTP impairment that persisted throughout the expression of LTP.

Other synaptic proteins including the NR2A- and NR2B-subunits of NMDAR and the GluA2 subunit of AMPAR, as well as pERK, pCREB (Fig 5B, 5C & 5D) were also down-regulated in WT hippocampi manifesting impaired LTP, thereby supporting that the PrP^Sc^ acute synaptotoxicity is not exclusively pre-synaptic. Although NMDAR are required for both pre- and post-synaptic functions, they are clearly most abundant post-synaptically [46]. Therefore, the reduced expression levels of NR2A and NR2B as well as GluA2 suggest post-synaptic disruptions in hippocampal glutamatergic synapses. The decreased expression levels of pERK and the transcription factor pCREB that activate pathways for production of synaptic proteins [47] further supports post-synaptic disruption. This suggests that pERK and pCREB are likely downstream effectors of PrP^Sc^ acute synaptotoxicity contributing to diminished expression of other synaptic proteins. Procaspase-3 was also down-regulated in hippocampi with impaired LTP (n = 5; Fig 5E & 5F), although the amount of active caspase 3 was normal (n = 5; Fig 5E & 5F), indicating that intracellular signalling cascades downstream of caspase-3 activation were probably normal, whereas mechanisms of generating procaspase-3 were conceivably disrupted. Importantly, expression levels of all these synaptic proteins were unaltered in hippocampal slices treated with dM1000 (Fig 5A–5F), correlating with the rescue of LTP and PPF ratio and despite persisting impairment of PTP. Moreover, post-synaptic markers such as PSD95 and Fyn remained unaltered in cM1000 (Fig 5C & 5D), suggesting that while PrP^Sc^ acutely disrupted post-synaptic function contributing to LTP impairment, it is less likely there was an acute loss of dendritic spine compartment volume over the very short time-course of our observations.

### PrP^Sc^ remains acutely synaptotoxic to PTP and LTP independent of PrP^C^ expression

**Fig 6:**
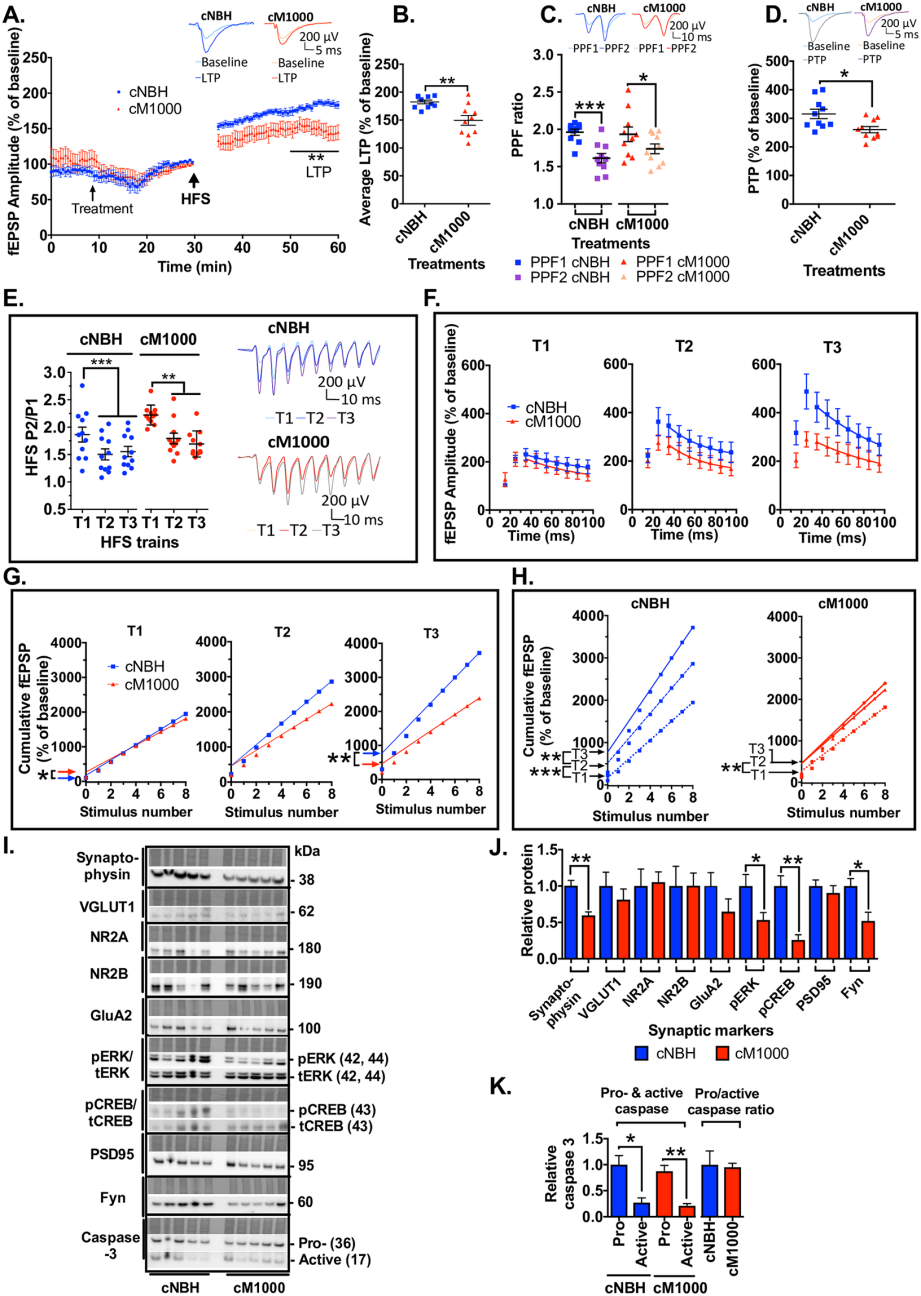
Acute PrP^Sc^ synaptotoxicity is largely PrP^C^ independent. Hippocampal slices derived from PrPo/o mice were used for these experiments. (A) cM1000 superfused over slices approximately 20 minutes prior to HFS trains caused significant impairment of LTP with (B) average LTP reduced by ~37 ± 12% (n = 10) compared with cNBH. (*p = 0*.*0046*). (C) A normal *Pr* during LTP in slices exposed to both cNBH and cM1000, where PPF2 (after treatment and LTP) became significantly reduced compared with the PPF1 (before treatment and LTP) (*p = 0*.*0003*). (D) PTP was significantly disrupted after exposure of slices to cM1000 relative to cNBH (*p = 0*.*0133*). (E) A normal increase in the *Pr* during HFS trains despite treatment with cM1000, demonstrated by a significant increase in the P1 to P2 ratio in T2 and T3 relative to T1. (F) A normal depletion rate of RRP after cM1000 exposure relative to cNBH across the three HFS trains, wherein the time constant of decay (Tau = 1/K) between P3 and P9 in each HFS train was not different between slices treated with cNBH and cM1000. (G) The size of RRP (determined by the Y-intercepts) in slices treated with cM1000 were modestly but significantly increased in T1 relative to slices treated with cNBH (cNBH: 173 ± 16, cM1000: 269 ± 15; *p = 0*.*0111*), demonstrating distinctive mechanisms of the acute synaptotoxicity in PrPo/o hippocampi; however, the RRP size of slices treated with cM1000 became significantly reduced in T3 relative to cNBH. (H) The RRP refill in slices treated with cM1000 (estimated by the increase in RRP size between trains) was normal between T1 and T2 (significantly increased from 169 ± 15 in T1 to 450 ± 28 in T2; *p = 0*.*0048*), but failed to significantly increase between T2 (450 ± 28) and T3 (461 ± 29) (G), thereby reflecting a significantly reduced RRP size. (I & J) Western blotting of PrPo/o hippocampi treated with cM1000 revealed reduced level of synaptophysin (*p = 0*.*0024*), but not VGLUT1. Exposure to cM1000 significantly reduced hippocampal pERK (*p = 0*.*0432*) and pCREB (*p = 0*.*0034*) relative to hippocampi treated with cNBH. Expression levels of NR2A and NR2B-containing NMDAR, and GluA2-containing AMPAR were unaffected by cM1000 treatment. PSD95 and Fyn levels were unaltered by cM1000 (*p = 0*.*0168*). (I & K) Pro-caspase 3 and production of its active form were unaltered in PrPo/o hippocampi treated with cM1000 relative to cNBH. (I) Molecular weight markers are provided at right. (A, C, D, E) Examples of raw fEPSP traces are provided as insets. Data are presented as ± SEM. *p<0.*05*, **p<0.*01*, ***p<0.*001*, ****p<0.*0001*.

Ongoing PrP^C^ expression is required for the sustained propagation of transmissible and neurotoxic PrP^Sc^ to cause prion disease [27, 31]. PrP^C^ has also been described as a receptor for transducing soluble, oligomeric Aβ42 synaptotoxicity [26]. To determine if PrP^Sc^ acute hippocampal synaptic disruption requires PrP^C^ expression, the synaptotoxicity of cM1000 was assessed using slices derived from 12-week-old PrPo/o mice. The degree of LTP impairment in these PrPo/o hippocampal slices following exposure to cM1000, was less marked but broadly comparable to that observed in WT slices (Fig 6A & 6B; see S5E Fig), thereby demonstrating that PrP^C^ expression is not crucial for the acute synaptotoxic mechanisms underlying disruption of LTP by PrP^Sc^. Notably though, in contrast to WT slices treated with cM1000 (Fig 4A), the PPF2 ratio significantly decreased following HFS trains, thereby mirroring what occurred with exposure to cNBH (Fig 6C; see S5G Fig) although PTP remained significantly impaired in PrPo/o slices following cM1000 treatment (Fig 6D; see S5F Fig). These findings suggest that any pre-synaptic dysfunction following HFS trains may also be at least partly PrP^C^ independent and that because the *Pr* was not significantly diminished during LTP expression the mechanisms of any PrP^Sc^ pre-synaptic dysfunction in PrPo/o slices are subtly but notably different compared with WT mice. Of further noteworthiness, the LTP, PTP, and PPF ratio in PrPo/o slices treated with cNBH were not affected compared with PrPo/o slices treated with aCSF (see S5 Fig), demonstrating no background synaptotoxicity from crude brain homogenates and importantly, the LTP, PTP, and PPF ratio obtained from PrPo/o and WT slices superfused with aCSF only were not different (see S5 Fig), in keeping with previous reports of no significant effect of the loss of PrP^C^ on the Schäffer collateral pathway synaptic functions in young PrPo/o mice [10].

### Different mechanisms appear to underlie PrP^Sc^ acute synaptotoxicity in PrPo/o hippocampal slices relative to WT slices

Given the likely fundamental role PrP^C^ plays in synaptic functions [7, 9], it is possible that the molecular components of the pre- and post-synaptic compartments are fundamentally altered to compensate the neuro-developmental absence of PrP^C^ in an attempt to maintain normal synaptic functions, thereby perhaps supporting the possibility of different or distinct mechanisms for any PrP^Sc^ acute synaptotoxicity. The disruption of PTP and LTP in PrPo/o hippocampal slices exposed to cM1000 suggested both pre- and post-synaptic impairments, respectively. The *Pr* during T2 and T3 of the HFS trains showed a normal decline comparable to that observed for cNBH (Fig 6E) and similar to what occurred after exposure of WT slices to cM1000 (Fig 4D). Importantly however, the RRP replenishment also appeared impaired in PrPo/o slices similar to that observed in WT slices (Fig 6G) leading to a significant reduction in RRP size during T3 (n = 11; Fig 6H), thereby probably disrupting PTP (n = 11; Fig 6D). Unexpectedly, the RRP during T1 was minimally but significantly larger in the PrPo/o slices exposed to cM1000 supporting the possibility of enhanced neurodevelopmental compensation to the absence of PrP^C^ made apparent in such slices upon exposure to PrP^Sc^ (n = 11; Fig 6H) explaining why a significant impairment of RRP replenishment was not apparent until T3.

Biochemical analysis of the cM1000 treated PrPo/o hippocampi revealed significantly decreased levels of synaptophysin (Fig 6I & 6J) akin to what was observed in WT slices (Fig 5A & 5D), further supporting a pre-synaptic component to the PrP^Sc^ acute synaptotoxicity in these slices and implying that the disruption of synaptophysin in WT hippocampal slices was probably PrP^C^ independent. In contrast, PrP^Sc^ exposure did not affect expression of VGLUT1 (Fig 6I & 6J), once again supporting that pre-synaptic functions in PrPo/o slices are probably less and differentially susceptible to PrP^Sc^ acute synaptotoxicity compared with WT slices. Additionally, expression levels of NR2A and NR2B and GluA2 remained unaffected by PrP^Sc^ in PrPo/o hippocampi, also supporting the likelihood of different post-synaptic pathophysiological mechanisms of PrP^Sc^ acute synaptotoxicity (Fig 6I & 6J) and implying that the down-regulation of VGLUT1, NR2A, NR2B, and GluA2 in WT hippocampal slices is PrP^C^ expression dependent. Nevertheless, importantly both pERK and pCREB were significantly reduced (Fig 6I & 6J) in PrPo/o slices, underscoring that although there are likely to be nuanced differences in the pathways sub-serving acute synaptic dysfunction in PrPo/o hippocampi, there appears to be overlap in the final effector pathway of post-synaptic dysfunction similar to WT hippocampi. PSD95 remained unaltered in PrPo/o hippocampi again supporting no net loss of the dendritic compartment associated with this acute synaptotoxicity over the very short time-frames of our experiments (Fig 6I & 6J). Interestingly, Fyn levels were significantly reduced in PrPo/o hippocampi after treatment with cM1000 (Fig 6I & 6J), supporting post-synaptic disruption in PrPo/o slices.

### WT and PrPo/o hippocampal slices display similar sensitivity to prion acute synaptotoxicity

**Fig 7:**
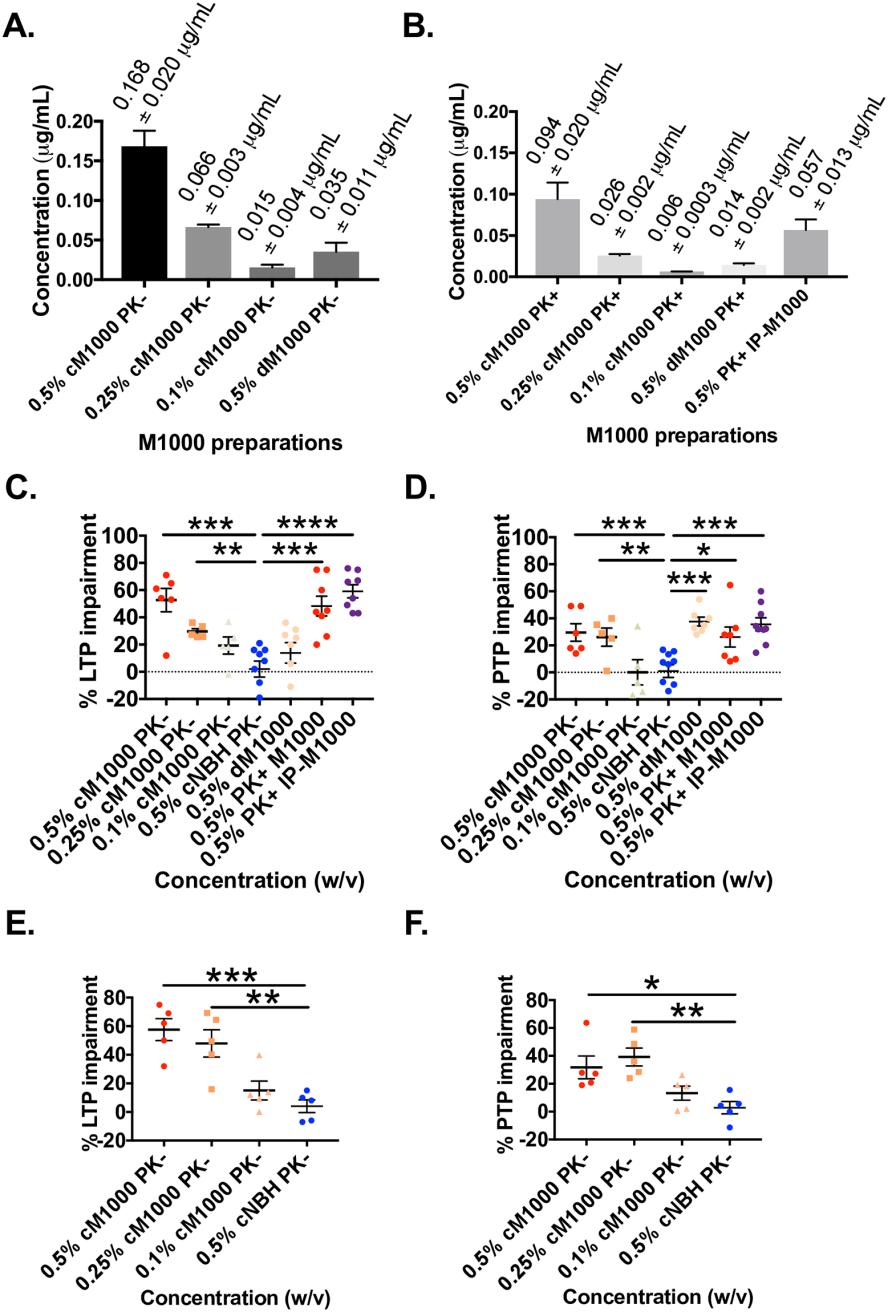
Crude M1000 brain homogenate acute synaptotoxicity dose-response relationships and estimation of total PrP and PK-resistant PrP^Sc^ in preparations used in electrophysiology experiments. A dilution series of recombinant full-length mouse PrP was western blotted (probed with 8H4 monoclonal antibody) and analysed with densitometry to generate a standard curve with aliquots of preparations utilised in electrophysiology experiments run on the same gel. (A) Quantification of levels of total PrP species (without PK treatment) in 0.5% (w/v), 0.25% (w/v), and 0.1% (w/v) cM1000, as well as in 0.5% dM1000 (PrP immuno-depleted cM1000). (B) Relative levels of at least modestly PK-resistant PrP^Sc^ (after treatment with 5μg/mL PK for 1 hour at 37°C) in 0.5% (w/v), 0.25% (w/v), and 0.1% (w/v) cM1000, as well as in 0.5% (w/v) PK+M1000 and 0.5% (w/v) PK+IP-M1000. (C & D) Percentage of LTP and PTP impairments (calculated as described in Methods) following exposure of 12-week old WT hippocampal slices to non-PK treated 0.5% (w/v), 0.25% (w/v), and 0.1% (w/v) cM1000 compared to 0.5% (w/v) cNBH controls (One-way ANOVA with Bonferroni correction for multiple comparisons [LTP: *p<0*.*0001*; PTP: *p<0*.*0001*]). In 0.5% (w/v) cM1000, LTP and PTP were significantly impaired by 53 ± 9% and 30 ± 6%, respectively. In 0.25% (w/v) cM1000, LTP and PTP were significantly impaired by 30 ± 2% and 26 ± 7%, respectively. In 0.1% (w/v) cM1000, LTP and PTP were not significantly impaired, reduced by only 19 ± 6% and 0.1 ± 9%, respectively. Percentages of LTP and PTP impairment in WT hippocampal slices following exposure to 0.5% dM1000, PK+M1000, and PK+IP-M1000 were also compared to 0.5% (w/v) cNBH controls (One-way ANOVA with Bonferroni correction for multiple comparisons [LTP: *p<0*.*0001*; PTP: *p<0*.*0039]*). In 0.5% (w/v) dM1000, the LTP was not impaired significantly (reduced by 14 ± 7%) while PTP was significantly impaired by 38 ± 3%. In 0.5% (w/v) PK+M1000, LTP and PTP were significantly impaired by 48 ± 7% and 26 ± 7%, respectively. In 0.5% (w/v) PK+IP-M1000, LTP and PTP were significantly impaired by 59 ± 5% and 36 ± 5%, respectively. (E & F) Percentage LTP and PTP impairments following exposure of 12-week old PrPo/o hippocampal slices to non-PK treated 0.5% (w/v), 0.25% (w/v), and 0.1% (w/v) cM1000 compared to 0.5% (w/v) cNBH controls (One-way ANOVA with Bonferroni correction for multiple comparisons [LTP: *p<0*.*0002*; PTP: *p<0*.*0027]*). In 0.5% (w/v) cM1000, LTP and PTP were significantly impaired by 58 ± 8% and 32 ± 8%, respectively. In 0.25% (w/v) cM1000, LTP and PTP were significantly impaired by 48 ± 10% and 39 ± 6%, respectively. In 0.1% (w/v) cM1000, LTP and PTP were not significantly impaired (reduced by only 15 ± 7% and 13 ± 5%, respectively). Data are presented as ± SEM. *p<0.*05*, **p<0.*01*, ***p<0.*001*, ****p<0.*0001*.

To determine if WT and PrPo/o hippocampal slices display similar sensitivity to prion acute synaptotoxicity, the dose-response relationships of LTP and PTP were analysed following exposure to a dilution series of cM1000 including 1% (w/v), 0.5% (w/v; as utilised previously), 0.25% (w/v) and 0.1% (w/v). In addition, the approximate level of abnormal or PK-resistant PrP^Sc^ in various preparations employed in our electrophysiological studies was determined. The 1% homogenates caused non-specific technical difficulties with electrophysiological testing precluding their use (S6A Fig). In 0.5% cM1000, there was ~0.168 ± 0.020 μg/mL total PrP including ~0.094 ± 0.020 μg/mL at least modestly PK-resistant PrP^Sc^ (n = 3; Fig 7A and 7B). In 0.25% cM1000 (n = 3), there was ~0.066 ± 0.003 μg/mL total PrP including ~0.026 ± 0.002 μg/mL at least modestly PK-resistant PrP^Sc^, while in 0.1% cM1000 there was ~0.015 ± 0.004 μg/mL total PrP with ~0.006 ± 0.0003 μg/mL at least modestly PK-resistant PrP^Sc^ (n = 3; Fig 7A and 7B).

For WT hippocampal slices, relative to cNBH controls, the LTP was significantly disrupted in a dose-dependent manner (*p<0*.*0001*; One-way ANOVA with Bonferroni correction for multiple comparisons) wherein non-PK digested 0.5% (n = 6) and 0.25% (n = 5) cM1000 significantly impaired LTP (0.5% *p<0*.0001, impaired by 53 ± 9%; 0.25% *p = 0*.*0195*, impaired by 30 ± 2%), while it was unaffected by the 0.1% cM1000 (n = 5; Fig 7C). Similarly, PTP in WT hippocampal slices was significantly impaired in a dose-dependent manner (*p = 0*.*0039*; One-way ANOVA with Bonferroni correction for multiple comparisons) following treatments with the same dilutions of cM1000 wherein both 0.5% (n = 6) and 0.25% (n = 5) cM1000 were significantly toxic to PTP (0.5% *p = 0*.*0073*, impaired by 30 ± 6%; 0.25% *p = 0*.*0270*, impaired by 26 ± 7%), while 0.1% cM1000 did not adversely affect PTP (n = 5; Fig 7D). Hence, these results demonstrated a correlation between the levels of PK-resistant PrP^Sc^ and the degree of LTP and PTP dysfunction with a threshold level of at least modestly PK-resistant PrP^Sc^ > ~0.006 μg/ml being toxic to PTP and although acknowledging a probable non-uniform immuno-depletion across the spectrum of PrP species in dM1000 a level >~0.014 μg/ml was toxic to LTP. Levels of at least modestly PK-resistant PrP^Sc^ in 0.5% PK+M1000 and PK+IP-M1000 that were significantly toxic to LTP and PTP were ~0.094 ± 0.020 μg/mL (n = 3) and 0.057 ± 0.013 μg/mL (n = 3), respectively.

For PrPo/o slices relative to cNBH controls, a similar dose-dependent decline in LTP and PTP was observed (n = 5 each; *p = 0*.*0027*; One-way ANOVA with Bonferroni correction for multiple comparisons). The 0.5% cM1000 reduced LTP significantly by 58 ± 8% (*p = 0*.*0003*), while 0.25% impaired LTP by 48 ± 10% (*p = 0*.*0018*); however, 0.1% cM1000 did not significantly disrupt LTP (Fig 7E). Parallel dose-dependent dysfunction was observed with PTP following exposure of PrPo/o hippocampal slices to 0.5%, 0.25%, and 0.1% cM1000, respectively (n = 5 each; *p = 0*.*0027*; One-way ANOVA with Bonferroni correction for multiple comparisons). Compared to cNBH controls, both 0.5% and 0.25% cM1000 significantly disrupted PTP (0.5% reduced PTP by 32 ± 8%, *p = 0*.*0135*; 0.25% reduced PTP by 39 ± 6%, *p = 0*.*0023*); however, 0.1% cM1000 was not significantly toxic to PTP (Fig 7F). Overall, the results were similar to those obtained in WT hippocampal slices, thereby implying that the prion acute synaptotoxicity in PrPo/o hippocampal slices reported in this current study was not due to an altered sensitivity.

## Discussion

Herein we report the acute synaptotoxicity of *ex vivo*, prion-containing preparations and provide important new molecular pathophysiological insights. Utilising a number of experimental approaches, our findings revealed that: (1) PrP^Sc^ (particularly at least modestly PK-resistant isoforms most likely as multimeric species) is directly synaptotoxic; (2) that the acute synaptotoxicity was similar across the two prion strains examined and appears independent of tissue source of prions when balanced for PrP^Sc^ levels; (3) the acute synaptotoxicity is not reliant on hippocampal PrP^C^ expression albeit with noteworthy molecular and electrophysiological differences in the presence or absence of PrP^C^; and (4) both pre- and post-synaptic functions are deleteriously affected, with the possibility of enhanced vulnerability of the former compartment.

Although previous studies have demonstrated clear correlations between the presence of PrP^Sc^ and the onset of neuropathological changes [38], disruption of LTP at the hippocampal CA1 region of mice inoculated with ME7 [14] and impairment of memory and learning [15], our studies offer compelling evidence for the direct synpatotoxicity of PrP^Sc^, especially PK-resistant species derived from brains of terminally sick animals. First, we demonstrated that modest PK treatment of *ex vivo* preparations (sufficient to digest all PrP^C^) did not attenuate the acute synaptotoxicity, with the impairment of LTP similar to that observed with cM1000. These protease digested preparations demonstrated substantial selective enrichment of PrP^Sc^ (see S3H Fig columns i & ii) analogous to that recently reported in another model assessing acute PrP^Sc^ synaptotoxicity [25]. Next, we demonstrated that immuno-depletion of PrP species from cM1000 substantially and proportionally mitigated the acute synaptotoxicity such that LTP was restored to levels not different to dNBH. Additionally, we demonstrated that the use of reconstituted, PK-eluted, immuno-precipitated PrP^Sc^ generated from crude brain preparations achieved full acute synaptotoxicity equivalent to that observed for cM1000, with additional biochemical characterisation of these preparations once again demonstrating significant selective enrichment of PrP^Sc^ (see S3H Fig columns iii & iv). Finally, size fractionation studies revealed that in addition to reducing total PrP species in dM1000, the spectrum of PK-resistant PrP^Sc^ species was altered and the levels of multimeric PrP^Sc^ species specifically decreased in dM1000 correlating with the rescue of LTP. Notably, the acute synaptotoxicity of cM1000 and PK+IP-M1000 clearly correlated with elevated levels of multimeric PK-resistant PrP^Sc^ species.

Our electrophysiology assay demonstrated that brains from terminally ill mice harbouring either M1000 or MU02 prions contain acutely synaptotoxic species. Interestingly, the degree and spectrum of synaptic dysfunction caused by these two different prion strains were indistinguishable, implying that the acute synaptotoxicity may be a generic property of PrP^Sc^ species and independent of the specific prion strain; the study of additional prion strains would confirm this speculation. Importantly, the degree of LTP impairment caused by both M1000 and MU02 strains was comparable to that recorded in the hippocampal CA1 region of ME7 scrapie-infected mice between 125 to 160 days post-inoculation [14], underscoring the biological validity of our findings. In further support of the biological relevance of our findings, the estimated level of misfolded or PK-resistant PrP^Sc^ in our 0.5% M1000 brain homogenates was ~0.094 ± 0.020 μg/mL, which despite methodological differences, falls well within the range of modestly (10 μg/ml) PK-resistant PrP^Sc^ levels determined by Mays et al. [48] in terminal whole brain homogenates of several prion animal models; however, uncertainty persists as to what precise levels would actually be found localised to synapses. Moreover, our results demonstrated a correlation between the levels of PK-resistant PrP^Sc^ and the degree of LTP and PTP dysfunction with a threshold level of at least modestly PK-resistant PrP^Sc^ > ~0.006 μg/ml appearing toxic to PTP while a level >~0.014 μg/ml appears toxic to LTP.

A recent study by Fang *et al* describes retraction and depletion of dendritic spines after 24 hours of exposure to PrP^Sc^ preparations [25]. In keeping with the importance of LTP in the generation and maintenance of dendritic spines, our observations may offer insights into the molecular pathogenesis underpinning at least some of the described dendritic spine morphological changes [12, 49, 50]. Though it is known that cellular expression is not required for uptake of PrP^Sc^ aggregates [51] and *in vivo* neurons not expressing PrP^C^ develop morphological abnormalities typical of prion disease with continued exposure to PrP^Sc^ over many weeks [52], in noteworthy contrast to this and our electrophysiology studies, Fang *et al* reported complete dependence on neuronal PrP^C^ expression for toxicity. There are several potential explanations, which from our cM1000 serial dilution studies do not relate to an altered sensitivity of PrPo/o hippocampal slices. Firstly, our model assessed synaptic dysfunction within a period of approximately 45 minutes following exposure to PrP^Sc^ containing preparations, whereas the time-frame for assessment in the study of Fang *et al* was at approximately 24 hours. The importance of ongoing neuronal PrP^Sc^ propagation to provide continued PrP^Sc^ exposure for effective pathogenesis is exemplified by the reported rapid and complete reversal of morphological, hippocampal CA1 region neurophysiological and behavioural abnormalities shortly after neuronal PrP^C^ expression is abrogated in the setting of established prion infection [53, 54]. The prompt reversibility of synaptic spine loss has also been reported in relation to AD models employing naturally secreted Aβ oligomers if the toxic species is completely removed [55]. Such observations support that the CNS has evolved powerful compensatory and recuperative mechanisms and it is highly likely that a single brief exposure to a synaptoxin only causes transient dysfunction that probably does not inevitably lead to sustained failure and loss of the synapse, especially if the level of toxin exposure is not extreme. Therefore we speculate in the Fang *et al* model that an additional approximately 23 hours in the absence of any *de novo* PrP^Sc^ production from endogenous PrP^C^ may have allowed effective activation of neuro-protective or adaptational responses in PrPo/o tissues, including progressive clearance or degradation of exogenous PrP^Sc^, and that persisting neuronal exposure above threshold levels is required to achieve sustained dendritic retraction and pruning when assessing synaptotoxicity over the longer 24 hour period. Secondly, other methodological differences, such as in the techniques for generating *ex vivo* PrP^Sc^ preparations (eg detergents plus serial ultracentrifugation versus IP plus PK digestion), the tissues used for toxicity studies (primary hippocampal neuronal cultures versus *ex vivo* hippocampal slices) and the primary metric for assessing toxicity (endogenous dendritic spine size/number versus induced LTP amplitudes), may collectively contribute to the observed discrepancies. Finally, and notwithstanding the aforementioned neurobiological and technical considerations, although LTP impairment was observed in PrPo/o slices, subtle but potentially important differences were observed in other facets of synaptic function, as well as in changes to key synaptic proteins. Namely, while PTP was also adversely affected by PrP^Sc^ preparations in addition to LTP in PrPo/o hippocampi, the PPF ratio showed a normal decline after LTP with VGLUT1, NR2A, NR2B, GluA2 and procaspase 3 levels unchanged. These discrepancies with WT hippocampi exposed to PrP^Sc^ suggest the possibility of PrP^C^-independent mechanistic pathways contribute to LTP and PTP impairment, which may not have been easily discerned in the setting of PrP^C^ expression and further underscore uncertainty of how such differences may relate to the retraction and loss of dendritic spines over a longer period of 24 hours.

The possible impact of age associated with the lack of PrP^C^ expression were important considerations in our studies. Because the absence of PrP^C^ has been reported to be linked with age-dependent impairment of hippocampal synaptic function in 8–15-month old mice [10], we mitigated this potentially confounding phenomenon by only using hippocampal slices from young PrPo/o mice. Notably, our findings reproduced those previously reported in a number of studies [10, 56, 57] with young PrPo/o mice exhibiting hippocampal CA1 synaptic functions congruent with aged-matched WT mice although contrary findings have been reported [58, 59], which may partly relate to variations in electrophysiology techniques employed. Also of interest, WT C57BL/6 mice 9–12 months old have been reported to express age-dependent deficits in learning following treatment with neurotoxic Aβ_1–42_ [60], thereby suggesting that older WT mice of this age may also be more susceptible to synaptic impairment associated with prion-infected preparations. Our studies however, revealed age-independent disruption of hippocampal synaptic function by cM1000 up to 11 months of age.

Synaptic physiology is complex and multi-facted making the achievement of precise understanding of pathophysiological changes through electrophysiological interrogation a considerable challenge. As part of validating the acute synaptotoxicity of PrP^Sc^, our detailed electrophysiological analyses suggest a possible enhanced pre-synaptic vulnerability, exemplified by the impairment of PTP, although contributions to LTP disruption from post-synaptic dysfunction seem likely as discussed below. A technical limitation of our assessments to probe pre-synaptic impairments was the lack of inclusion of specific GABA receptor antagonists. The 20 millisecond inter-stimulus intervals used to determine PPF ratios and the 10 millisecond intervals employed for HFS are very short, making contributions from GABA_B_ receptors quite unlikely but leaves open the possibility that GABA_A_ receptor related inhibitory post-synaptic potentials could have influenced some of the measured synaptic phenomena in ways we have not fully accounted for. Consequently, the absence of GABA_A_ receptor blockade in our experiments utilising fEPSP amplitudes to infer pre-synaptic pathophysiology allows for some potential inaccuracy in the derived measurements of *Pr* and RRP and precludes our studies from being definitive in this regard. Nevertheless and notwithstanding such caveats, to try to offer an understanding of the basis of the impairment of PTP, our analyses are consistent with impairment of action potential-dependent mechanisms of RRP replenishment, made obvious by the physiological stress imposed by repeated HFS trains used to induce PTP whereby there appeared to be a progressive deterioration of RRP replenishment leading to a significant reduction of RRP size and consequently a reduced PTP. In addition, because the *Pr* and the depletion rate of RRP during HFS trains appeared normal these could exacerbate the dysfunction of RRP replenishment [61] and the reduction of PTP. Further, this suggestion of pre-synaptic disruption was observed in WT and PrPo/o mice, supporting that PrP^C^ expression appears irrelevant to this dysfunction. The observation that immuno-depletion of total PrP species by ~77% only rescued LTP but not PTP with the ongoing PTP impairment also appearing to be due to deficiency of RRP replenishment leading to a reduction in RRP size, suggests that the modest amount of PK-resistant PrP^Sc^, including multimeric species, remaining after immuno-depletion appears sufficient to cause this disruption although contributions from a non-PrP^Sc^ factor cannot be completely excluded. These findings raise the possibility that the mechanisms responsible for RRP replenishment during repeated HFS trains appear highly vulnerable to PrP^Sc^ toxicity and more susceptible than LTP. The reduced PTP we observed appears discordant with results reported in a previous *in vivo* study of the ME7 prion strain in which PTP was reported as unaffected over the mid-incubation period [14]. We believe this apparent discrepancy is methodological in basis given that careful scrutiny of the relevant figure from this report reveals consistently lower amplitude initial PTPs in the prion infected mice similar to what we found. These authors measured PTP at a presumably varying time point during the first minute after HFS (with the PTP in non-prion infected mice decreasing rapidly after the first stimulation post-HFS) while we systematically utilised the first response immediately after serial HFS to estimate PTP.

The apparent disruption of the PPF ratio following HFS in WT slices is consistent with insufficient *Pr* during the maintenance of LTP suggesting pre-synaptic impairment following exposure to synaptotoxic PrP^Sc^ [62]. Additionally, expression levels of pre-synaptic markers, synaptophysin and VGLUT1, were consistently decreased in WT hippocampi manifesting disrupted LTP. Although synaptophsyin has been shown to be less important in neurotransmitter release [63], previous studies have uncovered that synaptophysin is significantly recruited for LTP expression and generally required for synaptic plasticity without involvement in *Pr* maintenance [64, 65]. Hence, this reduced level of synaptophysin is somehow directly concomitant with the impairment of LTP, but probably not via diminishing *Pr*. Noteworthy are previous reports of significantly reduced synaptophysin expression levels in the hippocampi of WT (C57BL/6) mice inoculated with three different prion strains: ME7, 79A, and 22L [66]. VGLUT1 instead plays substantial roles in the packaging of glutamate into pre-synaptic vesicles and maintaining normal *Pr*, wherein neurons lacking VGLUT1 have reduced *Pr* [67]. Therefore, we believe that the reduced expression levels of both synaptophysin and VGLUT1 directly contributed to the disruption of LTP, whereas only the loss of VGLUT1 was probably associated with our postulated impairment of *Pr* after LTP induction. Unexpectedly, we observed decreased synaptophysin levels but preserved VGLUT1 levels in PrPo/o slices correlating with the normal *Pr* observed following LTP. This suggests that the loss of VGLUT1 in WT hippocampi was PrP^C^ dependent, whereas the loss of synaptophysin in these hippocampi is PrP^C^ independent. The preservation of the *Pr* and PPF ratio following HFS in PrPo/o slices exposed to synaptotoxic PrP^Sc^ suggests that any pre-synaptic impairment associated with disruption of PTP is a primary pathophysiologic driver of the LTP dysfunction in both WT and PrPo/o slices. Nevertheless, because HFS also cause post-synaptic changes such as receptor activation, new receptor insertion and terminal expansion, which may collectively alter post-synaptic sensitivity to released neurotransmitters [12], contributions from post-synaptic disruptions are likely in the HFS-dependent synaptic dysfunction such as the impairment of LTP.

Variable transient disruption of the baseline during treatments of the hippocampal slices was observed in our study. Although we are not able to offer a definitive explanation for this minor technical issue, possible contributing factors, which are not mutually exclusive and may have occurred in varying combinations, include: i) slight variation in the temperature of some of the treatments; ii) slight variation in the pH of some of the treatments or slight pH change during the treatment; iii) slight variation in the depth of the treatment solution in each MEA recording chamber across experiments; and iv) minor mouse cohort variation in the sensitivity of hippocampal slices to the treatments. Another issue that could also contribute to variability, particularly in association with the above first three speculations is subtle variation in positioning of the superfusing solution inflow and outflow and the associated flow of solution within the MEA recording chamber. The MEA recording chambers are circular and this shape is known to allow some variation in cross-chamber solution flow as the solution can flow around the circular walls of the chamber rather than inevitably directly across the centre of the chamber; however, despite the occasional transient baseline disruption during treatments, there is no evidence this phenomenon impacted our recordings or results as HFS for elicitation of LTP and PTP was not undertaken until fEPSPs had returned to baseline for at least 5 minutes after the treatment. In addition, the acute synaptotoxic effects of *ex vivo* PrP^Sc^ preparations reported in our study were not linked to or affected by whether the baseline recording was temporarily disrupted (such as in Fig 1B cM1000 and 2H PK+M1000) or not disrupted (such as in Figures IE cMU02; 1K MoRK13-INF, and 2H PK+IP-M1000).

Notwithstanding controversies concerning the exact mechanisms underpinning hippocampal LTP, it is clear that LTP expression involves significant modifications of post-synaptic properties, particularly activation of extant post-synaptic NMDAR and externalization of new AMPAR [12, 68]. Our study demonstrated reduced expression levels of NR2A- and NR2B-containing NMDAR and GluA2-containing AMPAR in WT hippocampi expressing impaired LTP following exposure to synaptotoxic PrP^Sc^. Although these receptors can be expressed in both pre- and post-synaptic terminals [69, 70], their considerable numerical predominance post-synaptically supports that their overall loss was likely to be primarily post-synaptic [71]. NR2A/B-containing NMDAR are essential for hippocampal synaptic function and the expression of CA1 LTP [72]; therefore, their loss supports that the disruption of LTP in WT mice is at least partly dependent on NMDAR disruption. AMPAR also play a critical role in maintaining basal synaptic functions and most importantly in LTP expression, where new AMPAR are inserted into the post-synaptic membrane as part of LTP induction to enhance the function of active synapses and activate silent synapses [73, 74]. Because the acute PrP^Sc^ synaptotoxicity we observed was HFS-dependent, we postulate that the loss of AMPAR was mediated predominantly through silent synapses. Of likely relevance to our findings, parallel losses of NMDAR and AMPAR have been reported in the brains of AD patients [75] directly correlating with Aβ synaptotoxicity [76]. Unlike in WT slices, NR2A/B-containing NMDAR, and GluA2-containing AMPAR remained unaltered in PrPo/o hippocampi following exposure to synpatotoxic PrP^Sc^, implying different mechanisms are responsible for acute PrP^Sc^ synaptotoxicity in PrPo/o compared to WT slices. Importantly, this result infers that the disruptions of these receptors in WT mice are probably PrP^C^ dependent with the persistent impairment of LTP in PrPo/o hippocampi suggesting that the alterations of these glutamate receptors are not the sole determinant of PrP^Sc^ acute synaptotoxicity. This finding also suggests that PrPo/o mice may have higher innate resistance to PrP^Sc^ synaptotoxicity through mechanisms which mitigate acute synaptic dysfunction when studied over longer time frames than we allowed [77, 78].

Following exposure to PrP^Sc^ the key intracellular proteins pERK and pCREB involved in synaptic plasticity were also down-regulated in slices. Of note, both pERK and pCREB levels were significantly rescued following immuno-depletion of PrP, supporting a direct synaptotoxic effect of PrP^Sc^ on their function. Offering mechanistic pathway overlap in the disruption of LTP, these key intracellular synaptic proteins were also reduced in PrPo/o slices supporting that PrP^C^-independent impairment through different mechanisms occurs upstream of ERK and CREB activation. The sustained expression of NR2A and NR2B subunits in PrPo/o slices, while observing persistently reduced pERK and pCREB levels, supports the notion that activation of ERK and CREB during LTP induction and maintenance is likely to be independent of NMDAR activity in hippocampi of PrPo/o mice [79]. Although the activation of NMDAR is known to mediate the influx of Ca^2+^ during LTP induction, the activation of ERK and CREB has also been demonstrated to be independent of NMDAR activity but dependent on L-type voltage-gated calcium channel (L-VGCC) activation. PrP^C^ may be either directly or indirectly involved in Ca^2+^ influx via neuronal L-VGCCs [8, 80], and interact with Fyn to mediate Ca^2+^ dependent activation of ERK and CREB [81–83]. The absence of PrP^C^ in PrPo/o slices may increase the vulnerability of L-VGCCs to PrP^Sc^ disruption compared with WT mice.

Phosphorylation of ERK is essential for many synaptic functions such as activation of transcription factor CREB to produce more synaptic proteins. This pathway has been implicated in structural plasticity by which activation mediates formation of new dendritic spines and thereby contributes to LTP expression in *ex vivo* hippocampi [84] and memory formation in *vivo* [85]. Hence, the diminished levels of these two-key intracellular post-synaptic markers suggest that the acute PrP^Sc^ synaptotoxicity is likely to be associated with impaired production of new synaptic proteins with associated difficulties maintaining some spines and/or producing new spines. Of note, inhibition of ERK activity with MAPK/ERK kinase (MEK) inhibitors prevents dendritic spine growth and acutely disrupts LTP induction [68]. Additionally, pERK is directly involved in AMPAR insertion into the post-synaptic membrane following HFS, which is required for both enlarging synapses and activating silent synapses [47, 68, 73]. Hence, the reduced activation of pERK might have contributed to the down-regulation of AMPAR in WT mice but supports others factors contributing to this change in PrPo/o slices.

Overall, our observations offer new insights into the pathophysiology underlying the acute synaptic dysfunction of prion disease, generally aligning with reported synaptic disruptions demonstrated in other neurodegenerative diseases such as Alzheimer’s, Parkinson’s, and Huntington’s diseases, all of which consist of both pre-synaptic and post-synaptic impairments [86–88]. Our findings suggest an enhanced vulnerability of the pre-synaptic compartment, especially replenishment of the RRP of vesicles, to PrP^Sc^ acute synaptotoxicity although post-synaptic impairment is likely to contribute to the disruption of short and/or long-term synaptic plasticity. We have demonstrated that PrP^Sc^ derived from terminal disease brains, particularly modestly PK-resistant most likely multimeric species, are important direct synaptotoxins in prion disease, with the likelihood of PrP^C^-independent pathways contributing to acute PrP^Sc^ acute synaptotoxicity.

## Supporting information

S1 Table Primary and secondary antibodies used for immuno-blottings with their appropriate dilutions and comparable block buffer, which was either 5% Skim milk (Skm) or bovine serum albumin (BSA). (DOCX)

S1 Fig Methods and data analysis. (A) A schematic flow diagram of how the MEA experiment was done. (1) Mouse brain was sliced into hippocampal slices, (2) incubated at 32°C for an hour, (3) loaded onto the MEA (i) that was set-up with a perfusion system and (ii) stimulated the Schäffer collateral pathway through one of the micro-electrodes (black dots; the best aligned one) to evoke fEPSPs at the stratum radiatum of the CA1 region, and (4) the amplitudes of the fEPSPs were recorded as input-output (IO) curves and paired pulse facilitation (PPF) before (baseline) and after repetitive high frequency simulation (HFS) and the treatment of hippocampal slices with different preparations. (B) Examples of fEPSPs during baseline recording, PTP, LTP, and PPF. The PPF ratio was measured by employing two identical basal stimuli delivered at a 20 ms interval and recording the change in the elicited field excitatory post-synaptic potential (fEPSP); PPF1 recorded during the baseline and PPF2 after HFS and induction of long-term potentiation (LTP). (C) A series of individual fEPSP responses (P1 to P9) to each train (T) of the three HFS trains, with the ratio between P1 and P2 representing the initial probability of release (*Pr*) in each HFS train. (D) The slope of the curve through which the P3 fEPSP reduces to the P9 fEPSP signifies the rate of depletion of the readily releasable pool (RRP) during a single HFS train, with the slope measured as a time constant of decay (Tau = 1/K) employing a one-phase decay exponential function. (E) The size of RRP was estimated by back-extrapolating to the Y-intercept a linear fit equation based on the last 4 cumulative fEPSPs per train. During repeated trains (T1, T2 and T3) of HFS over a short period, the increases in RRP size from T1 to T2 and T2 to T3 represent the efficiency of RRP replenishment. (F) For the time-course PPF study without LTP induction, PPF was measured with basal stimulation before (PPF1A) and immediately after (PPF1B) exposure to either prion containing or control preparations; thereafter PPF1B was measured every 5 minutes for one hour.
(TIF)

S2 Fig Acute synaptotoxicity of crude *ex-vivo* PrP^Sc^ preparations. (A) Relative to the appropriate negative controls, there was a uniform and similar impairment of LTP (displayed as percentage change) caused by: 0.5% (w/v in aCSF) cM1000 (in both 12-week-old [related to Fig 1B & 1C] and 11-month-old hippocampal slices; [related to Fig 1G & 1H]); 0.5% (w/v in aCSF) cMU02 (in 12-week-old hippocampal slices; related to Fig 1E & 1F); and Two percent (w/v in aCSF) MoRK13-Inf (related to Fig 1K & 1L). To assess for potential non-PrP^Sc^ adverse synaptic effects in the various test preparations, LTP was compared to technical aCSF controls (aCSF only), with LTP (displayed as percentage of baseline field excitatory post-synaptic potential (fEPSP) amplitude over time [left panels] and as average LTP as a percentage of baseline [right panels]) not affected by: 0.5% (w/v in aCSF) cNBH in both (B) 12-week-old (related to Fig 1B & 1E) and (C) 11-month-old hippocampal slices (related to Fig 1H); (D) Two percent (w/v in aCSF) MoRK13-Un in 11-month-old hippocampal slices (related to Fig 1K). (E-H: first column i.) Normal I-O curves were obtained in all aCSF-only technical controls and (A-G, middle column ii.) the relevant negative controls whereby the I-O curves after LTP (I-O2) became significantly increased relative to the I-O curves before LTP (I-O1). In contrast, the I-O2 curves failed to significantly increase after exposure to PrP^Sc^ contained in: cM1000 (E: column iii—12-week-old hippocampal slices; F: column iii—11-month old mice hippocampal slices); cMU02 (G: column iii—12-week old mice hippocampal slices); MoRK13-Inf (H: column iii—11-month old mice hippocampal slices). Scatterplot: Student’s t test; I-O curves: Two-way ANOVA with repeated measures; mean ± SEM; *p<0.*05*, **p<0.*01*, ***p<0.*001*, ****p<0.*0001*.
(TIF)

S3 Fig Validation of the prion acute synaptotoxicity model. (A) Relative to the appropriate negative controls, there was a uniform and similar impairment of LTP (displayed as percentage change) caused by 0.5% (w/v; in aCSF) PK-treated cM1000 (PK+M1000) (in 12-week-old hippocampal slices; related to Fig 2E & 2F); and reconstituted PK-eluted PrP immuno-precipitated 0.5% (w/v) cM1000 (PK+IP-M1000) pellets (in 12-week-old hippocampal slices; related to Fig 2H & 2I). Importantly, immuno-depletion of PrP^Sc^ in 0.5% (w/v in aCSF) PrP immuno-depleted brain homogenate from cM1000 (dM1000) significantly rescued LTP to levels not different to the NBH control (related to Fig 2B & 2C). To assess for potential non-PrP^Sc^ synaptotoxicity in the various test preparations, LTP was compared to technical aCSF controls (aCSF only), with LTP (displayed as percentage of baseline field excitatory post-synaptic potential (fEPSP) amplitude over time [left panels] and as average LTP as a percentage of baseline [right panels]) not affected by (B) 0.5% (w/v in aCSF) PrP immuno-depleted NBH (dNBH) in 12-week-old hippocampal slices (related to Fig 1B); (C) PK treated 0.5% (w/v; in aCSF; with 5μg/ml PK) cNBH (PK+NBH) in 12-week-old hippocampal slices (related to Fig 1E); and (D) reconstituted PK-eluted immuno-precipitated 0.5% NBH (PK+IP-NBH) pellets in 12-week-old hippocampal slices (related to Fig 1H). The first five-minute fEPSP recordings following HFS trains have been omitted to assist clarity. (E-G) Normal I-O curves were demonstrated in aCSF (column i.) and negative controls (column ii.) wherein I-O2 became significantly increased relative to I-O1. Conversely, PK-treated cM1000 (PK+M1000) (E: column iii—12-week old mice hippocampal slices); and reconstituted, PK-eluted immuno-precipitated cM1000 brain homogenate (PK+IP-M1000) pellets (F: column iii—12-week old mice hippocampal slices) prevented I-O2 from becoming enhanced relative to I-O1. The only exception was following immuno-depletion of PrP (dM1000) wherein the I-O2 was normal compared with the controls (G: column iii—12-week old mice hippocampal slices). (H) Level of total protein (i, iii, v, vii) and PrP (ii, iv, vi, viii) in each *ex vivo* PrP preparation detected by coomassie stain and western blotting probed with 8H4 antibody. (I) Quantification of the total protein in each *ex vivo* PrP preparation. Relative to the total protein level in 0.5% (w/v) cM1000 without PK treatment, modest PK treatment (5μg/mL PK) of the 0.5% (w/v) cM1000 and IP-M1000 significantly reduced the total protein level to only 10% and 20% respectively, while containing substantial level of modestly PK-resistant PrP^Sc^ (H column ii & iv). Similarly, total proteins in 0.5% (w/v) cNBH and IP-NBH were significantly reduced to 20% and 30% by modest PK treatment compared with those in cNBH before PK treatment. However, no PrP detected by 8H4 antibody in modestly PK-treated NBH preparations (H column vi & viii). Densitometry analysis was performed as described in [Lewis, 2015 #248] Scatterplot: Student’s t test; I-O curves: Two-way ANOVA with repeated measures; mean ± SEM; *p<0.*05*, **p<0.*01*, ***p<0.*001*, ****p<0.*0001*.
(TIF)

S4 Fig Presynaptic functions. (A) Relative to aCSF-only technical controls where the paired pulse facilitation (PPF) ratio after LTP induction (PPF2) was significantly reduced compared with the PPF before LTP (PPF1), similar normal PPF results were obtained in the cNBH, MoRK13Un, dNBH, PK treated NBH, and PK-eluted IP-NBH controls (related to Fig 4A). (B) Synaptic disruption in the form of altered paired pulse facilitation (PPF) ratios (displayed as percentage change) was calculated after LTP (PPF2) relative to before LTP (PPF1). Relative to appropriate negative controls, all preparations containing PrP^Sc^ prevented reduction in the PPF ratio after LTP except dM1000, where the PPF ratio was significantly rescued (related to Fig 4A). (C) PTP displayed as a percentage of baseline fEPSP amplitude, was not affected by: Crude 0.5% (w/v in aCSF) brain homogenate from a normal brain (“sham”) inoculated mouse (cNBH) in both 12-week-old and 11-month-old hippocampal slices; crude 2.0% (w/v in aCSF) NBH “sham” uninfected RK13 cells lysate (MoRK13-Un) in 11-month-old hippocampal slices; 0.5% (w/v in aCSF) PrP immuno-depleted brain homogenate from a normal brain (“sham”) inoculated mouse (dNBH) in 12-week-old hippocampal slices; proteinase K (PK) treated 0.5% (w/v; in aCSF) cNBH (PK+NBH) in 12-week-old hippocampal slices; and reconstituted PK-eluted immunoprecipitated 0.5% NBH (PK+IP-NBH) pellets in 12-week-old hippocampal slices (related to Fig 4C). (D) Relative to the appropriate negative controls, all preparations containing PrP^Sc^, including the dM1000 caused similar PTP impairment (displayed as percentage change), thereby suggesting that PTP appears more sensitive to the acute synaptotoxic effects of PrP^Sc^ than LTP (related to Fig 4C). (E) Average baseline fEPSPs at every five minutes in hippocampal slices treated with aCSF, cNBH, and cM1000 (between the 10 and 15-minute time points) recorded for ~60 minutes. Scatterplot (A-D): mean ± SEM, Student’s t test; *p<0.*05*, **p<0.*01*, ***p<0.*001*, ****p<0.*0001*.
(TIF)

S5 Fig Additional electrophysiology results from PrPo/o hippocampal slices (related to Fig 6). (A-D) Analogous to what was observed in WT hippocampal slices, cNBH did not harbour non-PrP^Sc^ adverse synaptic effects on LTP (displayed as percentage of baseline fEPSP amplitude over time (A) or and as average LTP as a percentage of baseline (B), PTP (C) or PPF ratios after LTP induction (PPF2; D) in 12-week-old PrPo/o mice hippocampal slices relative to aCSF-only technical controls. (E & F) In comparison to cNBH, cM1000 impaired both LTP (E) and PTP (F) in 12-week old PrPo/o hippocampal slices (displayed as percentage change), with the changes comparable in degree to the disruption of LTP and PTP observed with 12-week-old WT hippocampal slices (related to Fig 6A, 6B & 6D). (G) In contrast, cM1000 did not impair the PPF ratio of 12-week-old PrPo/o hippocampal slices (displayed as percentage change) following LTP induction, with results not significantly different to cNBH (related to Fig 6C). (H) Relative to the aCSF-only technical control, cNBH did not disrupt the I-O curve after LTP expression (I-O2) compared with the I-O curve before LTP expression (I-O1) in 12-week-old PrPo/o hippocampal slices; however, cM1000 significantly impaired the enhancement of I-O2 relative to I-O1. Scatterplot: Student t test; Two-way ANOVA with repeated measures; mean ± SEM; *p<0.*05*, **p<0.*01*, ***p<0.*001*, ****p<0.*0001*.
(TIF)

S6 Fig The effects of 1% (w/v) versus 0.5% (w/v) crude normal brain homogenate (cNBH) on hippocampal synaptic functions (related to Fig 7). Relative to hippocampal slices from 12-week old wild type mice treated with aCSF, (A) slices treated with 1% (w/v) cNBH for five minutes following an eight to 10-minute stable baseline exhibited a disrupted baseline that did not recover back to normal baseline before the trains of high frequency stimulation (HFS), associated with disruption of the long-term potentiation (LTP). (B) However, hippocampal slices treated with 0.5% (w/v) cNBH did not affect the baseline and LTP.
(TIF)
