# Supplementary material for: Prion acute synaptotoxicity is largely driven by protease-resistant PrPSc species
Source: PLoS Pathog. 2018 Aug 8;14(8):e1007214. doi: 10.1371/journal.ppat.1007214 (PMC6101418; doi:10.1371/journal.ppat.1007214)
Supplement: S1 Table — (DOCX) [file ppat.1007214.s001.docx]

Supplementary Table 1**.** Antibodies and dilutions used for western blotting.

| **Primary antibody (company)** | **Dilution** | **Secondary antibody (HRP-conjugated)** | **Dilution** | **Block buffer and antibody diluent SkM or BSA)** |
| --- | --- | --- | --- | --- |
| 03R19 anti-PrP (Lawson et al., 2010) | 1:10000 | Anti-rabbit | 1:10000 | SkM |
| 8H4 (Abcam) | 1:5000 | Anti-mouse | 1:5000 | SkM |
| NR2A (Cell Signalling Technology; CST) | 1:2500 | Anti-rabbit | 1:5000 | SkM |
| NR2B (CST) | 1:2500 | Anti-rabbit | 1:5000 | SkM |
| GluA2 (CST) | 1:2500 | Anti-rabbit | 1:5000 | SkM |
| pERK (CST) | 1:2500 | Anti-rabbit | 1:5000 | BSA |
| ERK (CST) | 1:2500 | Anti-rabbit | 1:5000 | SkM |
| pCREB (CST) | 1:2500 | Anti-rabbit | 1:5000 | BSA |
| CREB (CST) | 1:2500 | Anti-mouse | 1:5000 | SkM |
| Synaptophysin (Millipore) | 1:2500 | Anti-mouse | 1:5000 | SkM |
| VGLUT1 (CST) | 1:2500 | Anti-rabbit | 1:5000 | SkM |
| Pro and active caspase 3 (Abcam) | 1:3000 | Anti-rabbit | 1:5000 | SkM |
