# Supplementary figures and images for: Prion acute synaptotoxicity is largely driven by protease-resistant PrPSc species
Source: PLoS Pathog. 2018 Aug 8;14(8):e1007214. doi: 10.1371/journal.ppat.1007214 (PMC6101418; doi:10.1371/journal.ppat.1007214)

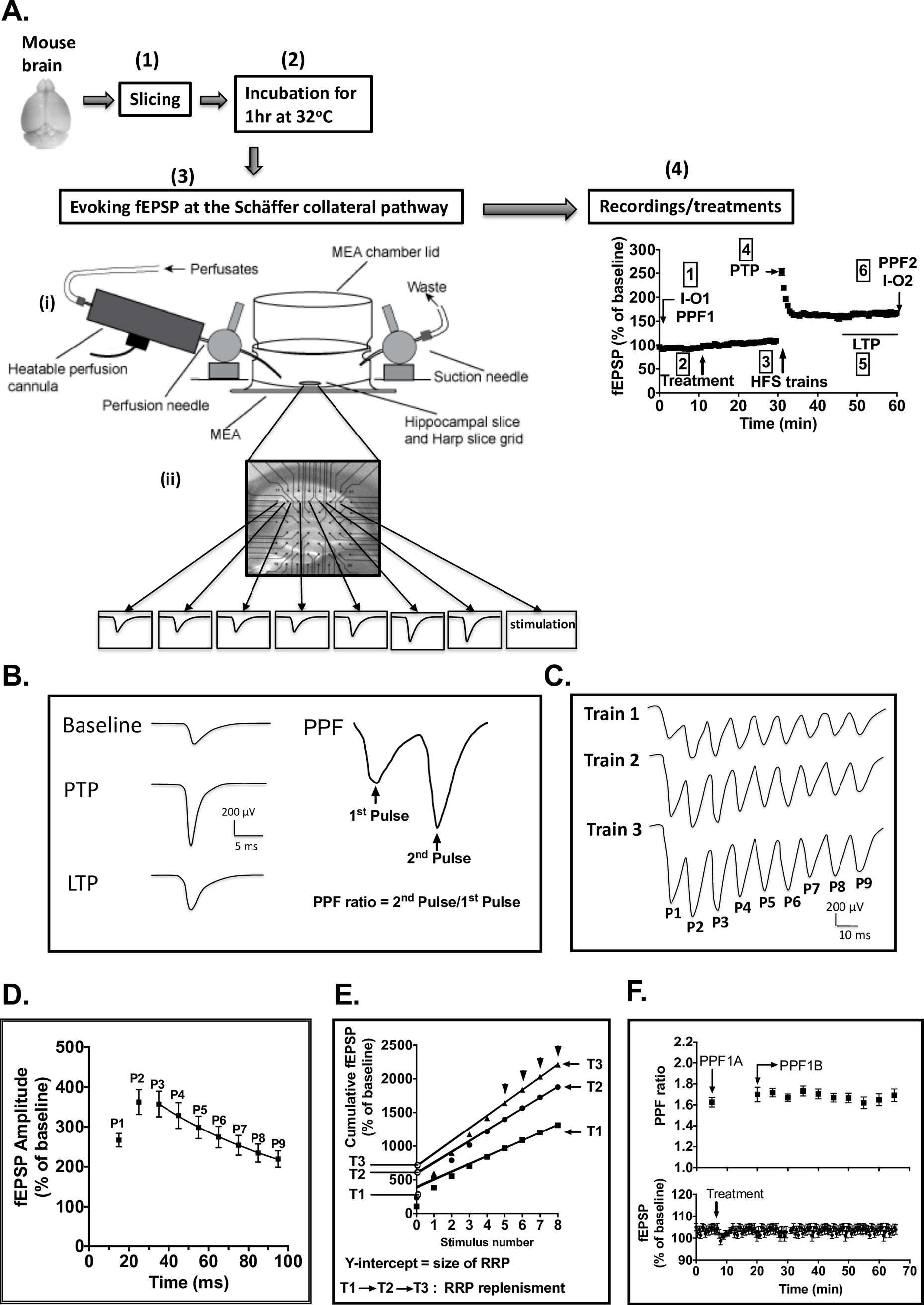

Supplement: S1 Fig — (A) A schematic flow diagram of how the MEA experiment was done. (1) Mouse brain was sliced into hippocampal slices, (2) incubated at 32°C for an hour, (3) loaded onto the MEA (i) that was set-up with a perfusion system and (ii) stimulated the Schäffer collateral pathway through one of the micro-electrodes (black dots; the best aligned one) to evoke fEPSPs at the stratum radiatum of the CA1 region, and (4) the amplitudes of the fEPSPs were recorded as input-output (IO) curves and paired pulse facilitation (PPF) before (baseline) and after repetitive high frequency simulation (HFS) and the treatment of hippocampal slices with different preparations. (B) Examples of fEPSPs during baseline recording, PTP, LTP, and PPF. The PPF ratio was measured by employing two identical basal stimuli delivered at a 20 ms interval and recording the change in the elicited field excitatory post-synaptic potential (fEPSP); PPF1 recorded during the baseline and PPF2 after HFS and induction of long-term potentiation (LTP). (C) A series of individual fEPSP responses (P1 to P9) to each train (T) of the three HFS trains, with the ratio between P1 and P2 representing the initial probability of release (Pr) in each HFS train. (D) The slope of the curve through which the P3 fEPSP reduces to the P9 fEPSP signifies the rate of depletion of the readily releasable pool (RRP) during a single HFS train, with the slope measured as a time constant of decay (Tau = 1/K) employing a one-phase decay exponential function. (E) The size of RRP was estimated by back-extrapolating to the Y-intercept a linear fit equation based on the last 4 cumulative fEPSPs per train. During repeated trains (T1, T2 and T3) of HFS over a short period, the increases in RRP size from T1 to T2 and T2 to T3 represent the efficiency of RRP replenishment. (F) For the time-course PPF study without LTP induction, PPF was measured with basal stimulation before (PPF1A) and immediately after (PPF1B) exposure to either prion containing or c [file ppat.1007214.s002.tif]

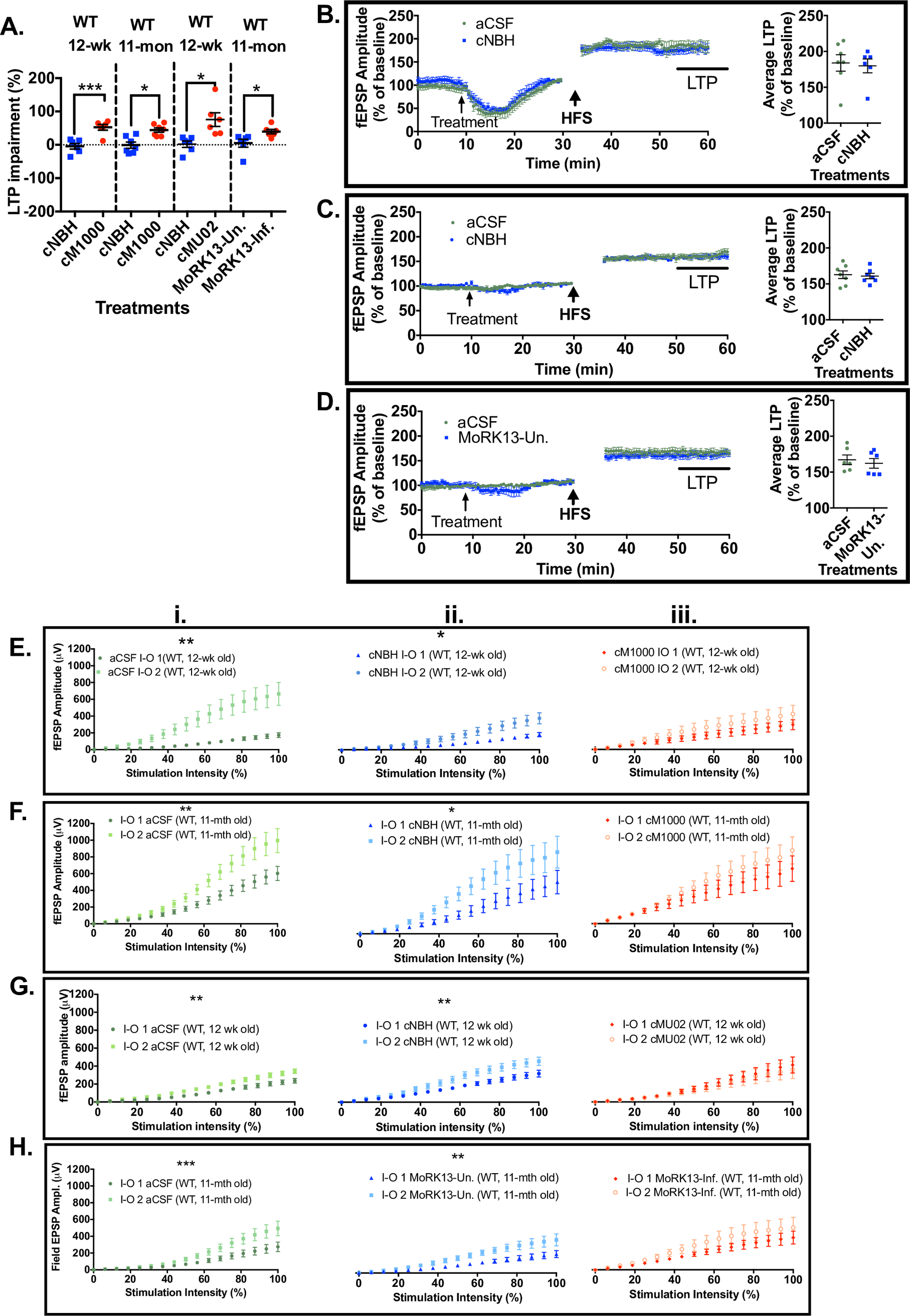

Supplement: S2 Fig — (A) Relative to the appropriate negative controls, there was a uniform and similar impairment of LTP (displayed as percentage change) caused by: 0.5% (w/v in aCSF) cM1000 (in both 12-week-old [related to Fig 1B & 1C] and 11-month-old hippocampal slices; [related to Fig 1G & 1H]); 0.5% (w/v in aCSF) cMU02 (in 12-week-old hippocampal slices; related to Fig 1E & 1F); and Two percent (w/v in aCSF) MoRK13-Inf (related to Fig 1K & 1L). To assess for potential non-PrPSc adverse synaptic effects in the various test preparations, LTP was compared to technical aCSF controls (aCSF only), with LTP (displayed as percentage of baseline field excitatory post-synaptic potential (fEPSP) amplitude over time [left panels] and as average LTP as a percentage of baseline [right panels]) not affected by: 0.5% (w/v in aCSF) cNBH in both (B) 12-week-old (related to Fig 1B & 1E) and (C) 11-month-old hippocampal slices (related to Fig 1H); (D) Two percent (w/v in aCSF) MoRK13-Un in 11-month-old hippocampal slices (related to Fig 1K). (E-H: first column i.) Normal I-O curves were obtained in all aCSF-only technical controls and (A-G, middle column ii.) the relevant negative controls whereby the I-O curves after LTP (I-O2) became significantly increased relative to the I-O curves before LTP (I-O1). In contrast, the I-O2 curves failed to significantly increase after exposure to PrPSc contained in: cM1000 (E: column iii—12-week-old hippocampal slices; F: column iii—11-month old mice hippocampal slices); cMU02 (G: column iii—12-week old mice hippocampal slices); MoRK13-Inf (H: column iii—11-month old mice hippocampal slices). Scatterplot: Student’s t test; I-O curves: Two-way ANOVA with repeated measures; mean ± SEM; *p<0.05, **p<0.01, ***p<0.001, ****p<0.0001. (TIF) [file ppat.1007214.s003.tif]

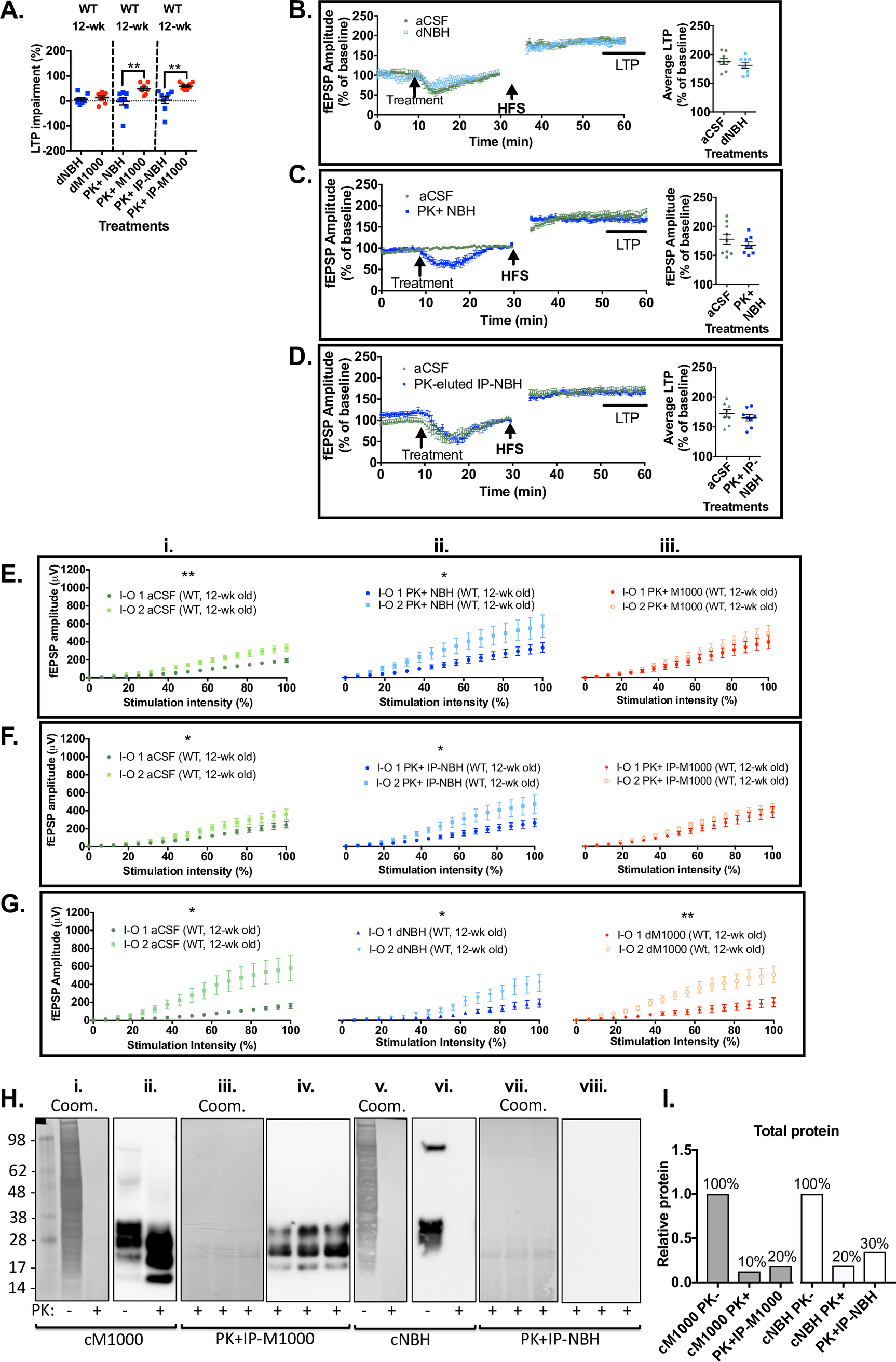

Supplement: S3 Fig — (A) Relative to the appropriate negative controls, there was a uniform and similar impairment of LTP (displayed as percentage change) caused by 0.5% (w/v; in aCSF) PK-treated cM1000 (PK+M1000) (in 12-week-old hippocampal slices; related to Fig 2E & 2F); and reconstituted PK-eluted PrP immuno-precipitated 0.5% (w/v) cM1000 (PK+IP-M1000) pellets (in 12-week-old hippocampal slices; related to Fig 2H & 2I). Importantly, immuno-depletion of PrPSc in 0.5% (w/v in aCSF) PrP immuno-depleted brain homogenate from cM1000 (dM1000) significantly rescued LTP to levels not different to the NBH control (related to Fig 2B & 2C). To assess for potential non-PrPSc synaptotoxicity in the various test preparations, LTP was compared to technical aCSF controls (aCSF only), with LTP (displayed as percentage of baseline field excitatory post-synaptic potential (fEPSP) amplitude over time [left panels] and as average LTP as a percentage of baseline [right panels]) not affected by (B) 0.5% (w/v in aCSF) PrP immuno-depleted NBH (dNBH) in 12-week-old hippocampal slices (related to Fig 1B); (C) PK treated 0.5% (w/v; in aCSF; with 5μg/ml PK) cNBH (PK+NBH) in 12-week-old hippocampal slices (related to Fig 1E); and (D) reconstituted PK-eluted immuno-precipitated 0.5% NBH (PK+IP-NBH) pellets in 12-week-old hippocampal slices (related to Fig 1H). The first five-minute fEPSP recordings following HFS trains have been omitted to assist clarity. (E-G) Normal I-O curves were demonstrated in aCSF (column i.) and negative controls (column ii.) wherein I-O2 became significantly increased relative to I-O1. Conversely, PK-treated cM1000 (PK+M1000) (E: column iii—12-week old mice hippocampal slices); and reconstituted, PK-eluted immuno-precipitated cM1000 brain homogenate (PK+IP-M1000) pellets (F: column iii—12-week old mice hippocampal slices) prevented I-O2 from becoming enhanced relative to I-O1. The only exception was following immuno-depletion of PrP (dM1000) wherein the I-O2 was normal compared with the [file ppat.1007214.s004.tif]

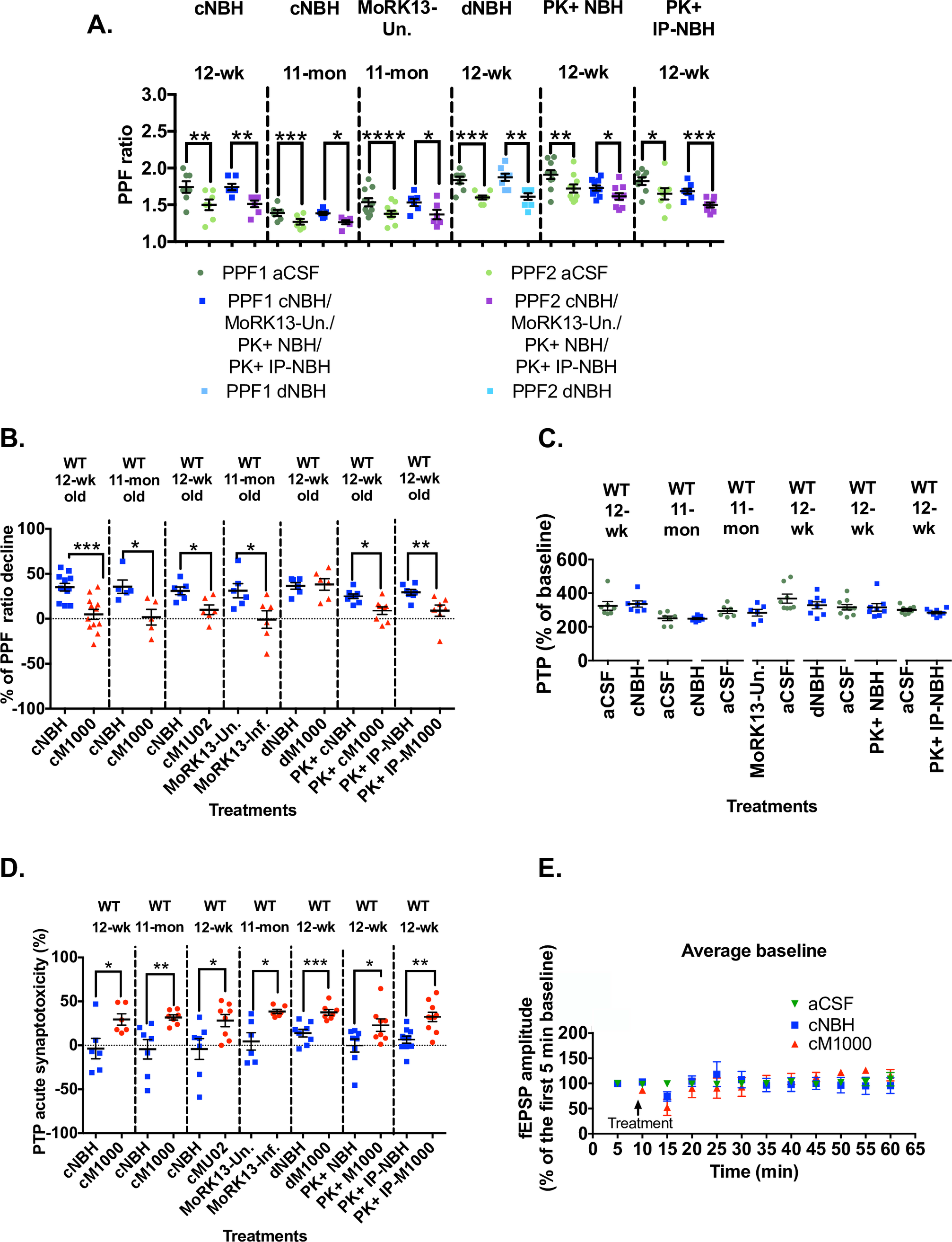

Supplement: S4 Fig — (A) Relative to aCSF-only technical controls where the paired pulse facilitation (PPF) ratio after LTP induction (PPF2) was significantly reduced compared with the PPF before LTP (PPF1), similar normal PPF results were obtained in the cNBH, MoRK13Un, dNBH, PK treated NBH, and PK-eluted IP-NBH controls (related to Fig 4A). (B) Synaptic disruption in the form of altered paired pulse facilitation (PPF) ratios (displayed as percentage change) was calculated after LTP (PPF2) relative to before LTP (PPF1). Relative to appropriate negative controls, all preparations containing PrPSc prevented reduction in the PPF ratio after LTP except dM1000, where the PPF ratio was significantly rescued (related to Fig 4A). (C) PTP displayed as a percentage of baseline fEPSP amplitude, was not affected by: Crude 0.5% (w/v in aCSF) brain homogenate from a normal brain (“sham”) inoculated mouse (cNBH) in both 12-week-old and 11-month-old hippocampal slices; crude 2.0% (w/v in aCSF) NBH “sham” uninfected RK13 cells lysate (MoRK13-Un) in 11-month-old hippocampal slices; 0.5% (w/v in aCSF) PrP immuno-depleted brain homogenate from a normal brain (“sham”) inoculated mouse (dNBH) in 12-week-old hippocampal slices; proteinase K (PK) treated 0.5% (w/v; in aCSF) cNBH (PK+NBH) in 12-week-old hippocampal slices; and reconstituted PK-eluted immunoprecipitated 0.5% NBH (PK+IP-NBH) pellets in 12-week-old hippocampal slices (related to Fig 4C). (D) Relative to the appropriate negative controls, all preparations containing PrPSc, including the dM1000 caused similar PTP impairment (displayed as percentage change), thereby suggesting that PTP appears more sensitive to the acute synaptotoxic effects of PrPSc than LTP (related to Fig 4C). (E) Average baseline fEPSPs at every five minutes in hippocampal slices treated with aCSF, cNBH, and cM1000 (between the 10 and 15-minute time points) recorded for ~60 minutes. Scatterplot (A-D): mean ± SEM, Student’s t test; *p<0.05, **p<0.01, ***p<0.001, ****p<0.0001. (TI [file ppat.1007214.s005.tif]

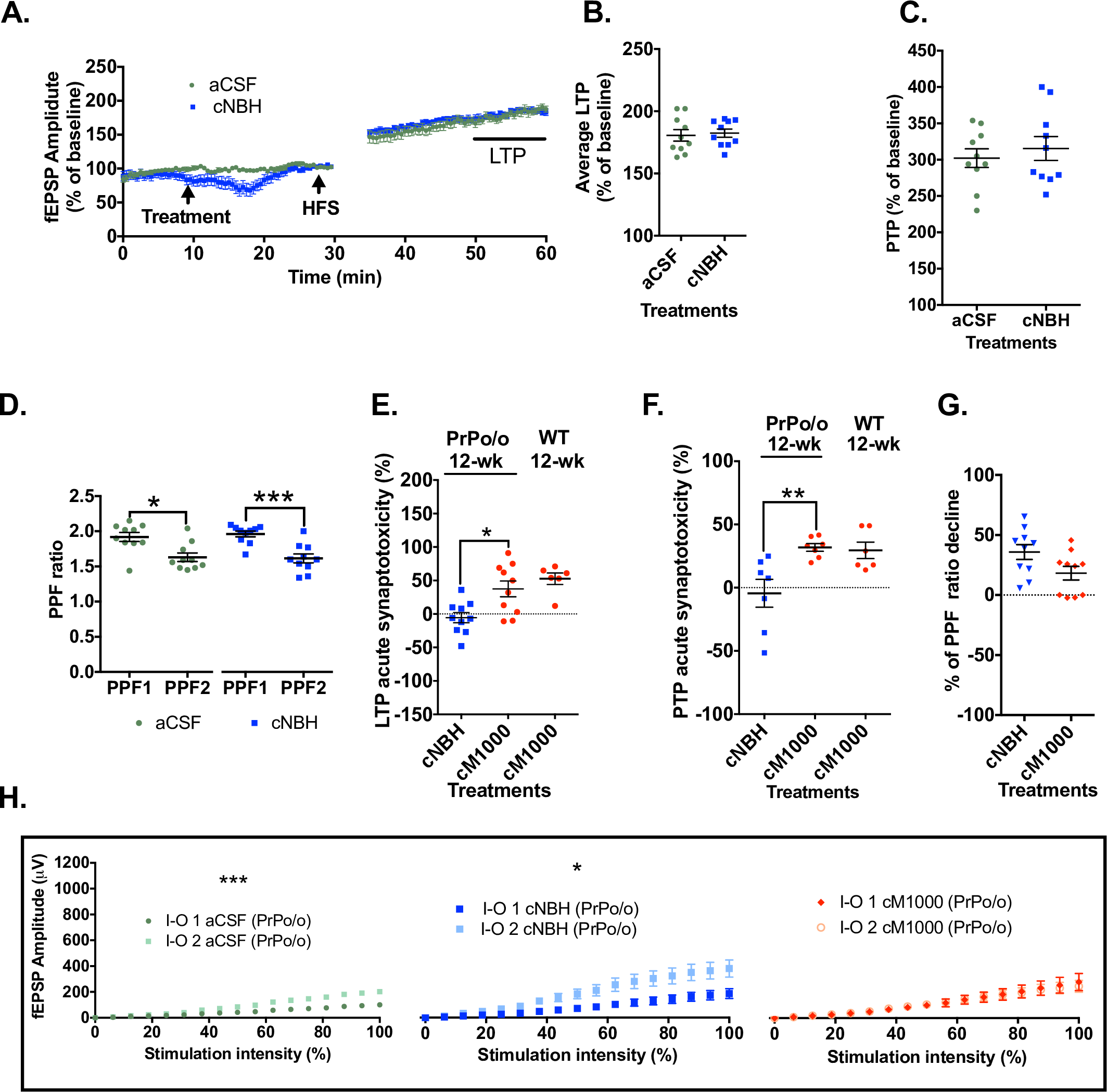

Supplement: S5 Fig — (A-D) Analogous to what was observed in WT hippocampal slices, cNBH did not harbour non-PrPSc adverse synaptic effects on LTP (displayed as percentage of baseline fEPSP amplitude over time (A) or and as average LTP as a percentage of baseline (B), PTP (C) or PPF ratios after LTP induction (PPF2; D) in 12-week-old PrPo/o mice hippocampal slices relative to aCSF-only technical controls. (E & F) In comparison to cNBH, cM1000 impaired both LTP (E) and PTP (F) in 12-week old PrPo/o hippocampal slices (displayed as percentage change), with the changes comparable in degree to the disruption of LTP and PTP observed with 12-week-old WT hippocampal slices (related to Fig 6A, 6B & 6D). (G) In contrast, cM1000 did not impair the PPF ratio of 12-week-old PrPo/o hippocampal slices (displayed as percentage change) following LTP induction, with results not significantly different to cNBH (related to Fig 6C). (H) Relative to the aCSF-only technical control, cNBH did not disrupt the I-O curve after LTP expression (I-O2) compared with the I-O curve before LTP expression (I-O1) in 12-week-old PrPo/o hippocampal slices; however, cM1000 significantly impaired the enhancement of I-O2 relative to I-O1. Scatterplot: Student t test; Two-way ANOVA with repeated measures; mean ± SEM; *p<0.05, **p<0.01, ***p<0.001, ****p<0.0001. (TIF) [file ppat.1007214.s006.tif]

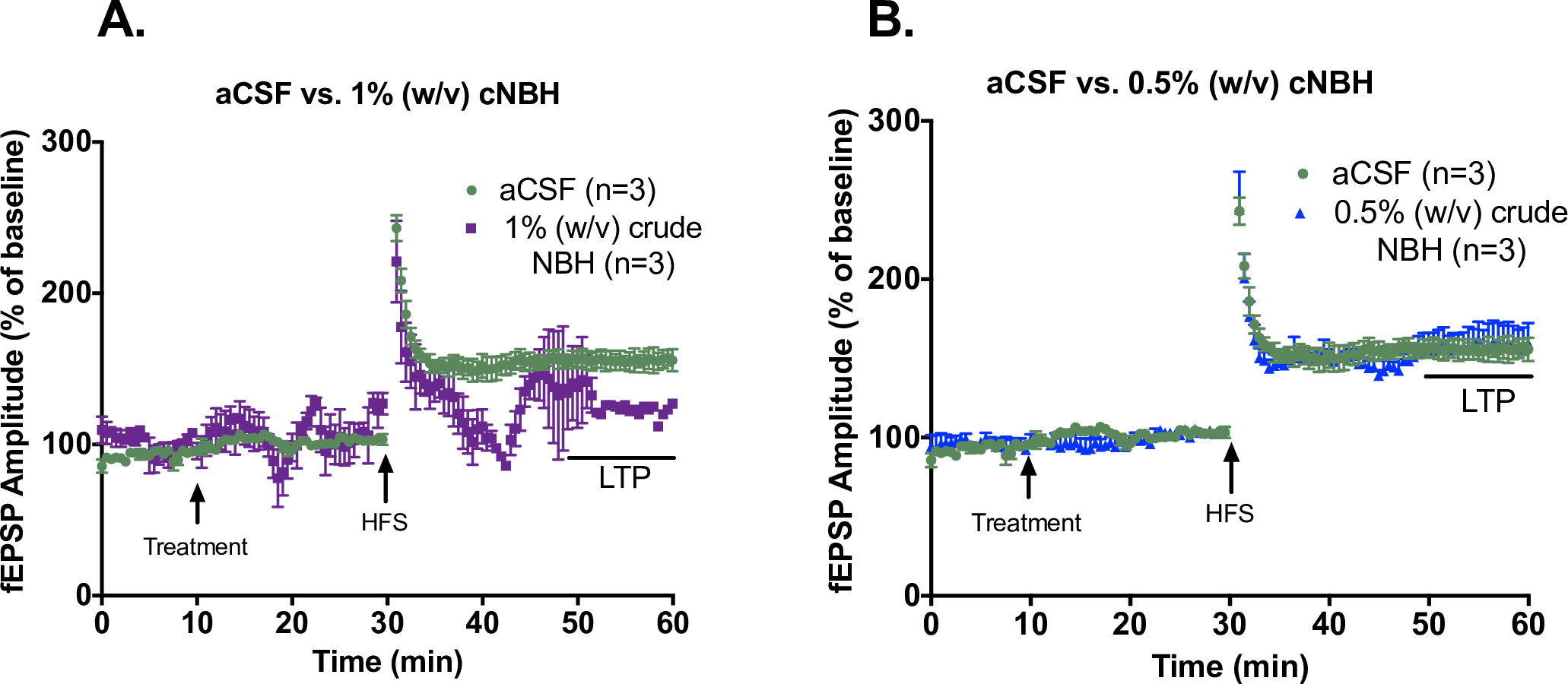

Supplement: S6 Fig — Relative to hippocampal slices from 12-week old wild type mice treated with aCSF, (A) slices treated with 1% (w/v) cNBH for five minutes following an eight to 10-minute stable baseline exhibited a disrupted baseline that did not recover back to normal baseline before the trains of high frequency stimulation (HFS), associated with disruption of the long-term potentiation (LTP). (B) However, hippocampal slices treated with 0.5% (w/v) cNBH did not affect the baseline and LTP. (TIF) [file ppat.1007214.s007.tif]
